# Single-cell transcriptomics identifies Mcl-1 as a target for senolytic therapy in cancer

**DOI:** 10.1038/s41467-022-29824-1

**Published:** 2022-04-21

**Authors:** Martina Troiani, Manuel Colucci, Mariantonietta D’Ambrosio, Ilaria Guccini, Emiliano Pasquini, Angelica Varesi, Aurora Valdata, Simone Mosole, Ajinkya Revandkar, Giuseppe Attanasio, Andrea Rinaldi, Anna Rinaldi, Marco Bolis, Pietro Cippà, Andrea Alimonti

**Affiliations:** 1 Institute of Oncology Research (IOR), Oncology Institute of Southern Switzerland (IOSI), CH6500 Bellinzona, Switzerland; 2 Università della Svizzera Italiana, CH6900 Lugano, Switzerland; 3 Bioinformatics Core Unit, Swiss Institute of Bioinformatics, TI, 6500 Bellinzona, Switzerland; 4 Faculty of Biology and Medicine, University of Lausanne UNIL, CH1011 Lausanne, Switzerland; 5 Institute of Molecular Health Sciences, ETH Zurich, CH8093 Zurich, Switzerland; 6 Massachusetts General Hospital Cancer Center, Harvard Medical School, Charlestown, MA 02129 USA; 7 Department of Medicine, Division of Nephrology, Ente Ospedaliero Cantonale, Lugano, Switzerland; 8 Laboratories for Translational Research, Ente Ospedaliero Cantonale, Bellinzona, Switzerland; 9 Computational Oncology Unit, Department of Oncology, Istituto di Ricerche Farmacologiche ‘Mario Negri’ IRCCS, 20156 Milano, Italy; 10 Department of Health Sciences and Technology (D-HEST) ETH Zurich, 8093 Zurich, CH Switzerland; 11 Department of Medicine & Veneto Institute of Molecular Medicine, University of Padova, Padova, Italy

**Keywords:** Bioinformatics, Cancer models, Senescence, Cancer genomics, Targeted therapies

## Abstract

Cells subjected to treatment with anti-cancer therapies can evade apoptosis through cellular senescence. Persistent senescent tumor cells remain metabolically active, possess a secretory phenotype, and can promote tumor proliferation and metastatic dissemination. Removal of senescent tumor cells (senolytic therapy) has therefore emerged as a promising therapeutic strategy. Here, using single-cell RNA-sequencing, we find that senescent tumor cells rely on the anti-apoptotic gene Mcl-1 for their survival. Mcl-1 is upregulated in senescent tumor cells, including cells expressing low levels of Bcl-2, an established target for senolytic therapy. While treatment with the Bcl-2 inhibitor Navitoclax results in the reduction of metastases in tumor bearing mice, treatment with the Mcl-1 inhibitor S63845 leads to complete elimination of senescent tumor cells and metastases. These findings provide insights on the mechanism by which senescent tumor cells survive and reveal a vulnerability that can be exploited for cancer therapy.

## Abstract (web summary)

Cell senescence remains a barrier to tumor elimination in many cancers. Here, the authors use single cell RNA-seq to identify a role for Mcl-1 in senescent cell survival, and show that Mcl-1 inhibition may be an effective therapeutic strategy.

## Introduction

Cells subjected to elevated stress such as treatment with anti-cancer therapies can react in two ways: either die or remain suspended in between life and death, a condition known as cellular senescence^1–4^. Cellular senescence is a stable cell growth arrest that occurs in tumor cells subjected to different insults including treatment with chemo-radiotherapy or targeted therapies^1,2,4–6^. Although arrested, senescent tumor cells remain metabolically active and secrete in the tumor microenvironment a variety of cytokines and inflammatory factors, known as the senescence-associated secretory phenotype (SASP)^1,2,5,7^. Several findings in vivo demonstrate that senescence limits tumor progression by arresting cancer cells and promoting immune surveillance^4,5,7^. However, after an initial beneficial phase, persistent senescent tumor cells can promote tumor growth, migration, and even metastases^2,6,7^. These deleterious cancer phenotypes have been described, particularly in tumor cells treated with therapy-induced senescence (TIS). Cancer cells, that have entered TIS can, in fact, escape the senescent growth arrest and acquire more aggressive phenotypes associated with increased stemness and drug resistance^8,9^. Moreover, the SASP of senescent tumor cells can promote the proliferation and migration of neighboring tumor cells. Thus, the selective removal of senescent cells, known as senolytic therapy, has been proposed as a strategy to improve the efficacy of currently available treatments in tumors where immunosuppression hinders the clearance of senescent cells^3,6,10^. However, several reports examining the effectiveness of different senolytic agents in combination with TIS in cancer models have raised significant issues and potential concerns. Navitoclax, a BH3 mimetic that binds and neutralizes Bcl-2 and Bcl-xL, is the most promising of this class of compounds^11–13^. However, the efficacy of Navitoclax depends on the genetic background of the senescent tumor cells, being only partially effective in some tumor types. Moreover, Bcl-2 inhibitor treatment is associated to severe toxicity that limits their clinical application when used either alone or in combination with TIS^6,13–16^. Single-cell RNA-sequencing (scRNA-seq) technology has created unprecedented opportunities to simultaneously assess thousands of cells within a sample, enabling the evaluation of heterogeneity among tumor cells^17,18^. Furthermore, scRNA-seq provides unique opportunities to assess the regulation, evolution and interaction of individual cells and the identification of specific cell types^17,19^. Although scRNA-seq has been increasingly adopted, its application to senescence in cancer is still limited.

In this paper, we show that scRNA-seq is a reliable approach to define, characterize, and identify common vulnerabilities in senescent tumor cells of different genetic backgrounds to develop more specific senolytics to be used in combination with standard of therapy in future clinical trials. In this work by using single cells analysis, we find that senescent prostate tumor cells are heterogenous, but rely on common pro-survival pathways. Interestingly, we find that the Myeloid Cell Leukemia 1 (Mcl-1) is the most expressed anti-apoptotic gene in senescent tumor cells, being overexpressed even in Bcl-2-negative senescent cancer cells. In line with these findings, pharmacological inhibition of Mcl-1 eliminates senescent prostate tumor cells, blocking tumor progression and metastases.

## Results

### Identification of senescent prostate cancer cells across different mouse models

**Fig. 1:**
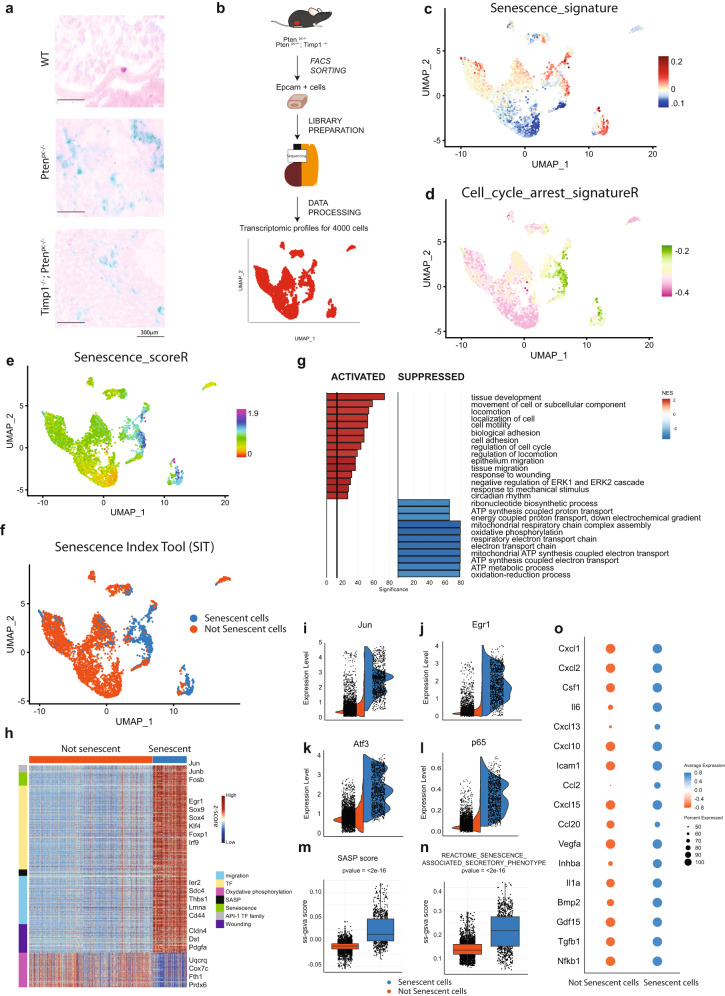
Overview of the identification of senescent cells in prostate cancer. **a** Representative images of SA-β-Gal in *WT*, *Pten*^*pc−/−*^ and *Pten*^*pc−/−*^*; Timp1*^*−/−*^ genotypes (Scale bar 300 μm). Data are representative of two independent experiments. **b** Schematic representation of single cells isolation and sequencing (*n* = 4 mice). **c** UMAP plot of cancer epithelial cells colored by Senescence_signature. **d** UMAP plot of cancer epithelial cells colored by Cell_cycle_arrest_signatureR. **e** UMAP plot of cancer epithelial cells colored by Senescence_scoreR. **f** UMAP plot of cancer epithelial cells showing senescent cells (blue) and not senescent cells (orange) found through SIT. **g** Bar plot showing enrichment pathway analysis of senescent cells compared to not senescent cells. gseGO function results: Weighted Kolmogorov Smirnov (WKS) test followed by FDR correction. **h** Heatmap of differentially expressed genes between senescent and not senescent cells. Row annotation showing the functions of different genes (migration, SASP, wounding, TF). Wilcoxon Rank Sum test followed by FDR correction. **i** Violin plot of the most upregulated gene in senescent cells: *Jun*. **j**, **k** Violin plot of key transcription factors involved in senescence transcriptional program: *Egr1*, and *Atf3*. **l** Violin plot showing expression of *p65/RelA* in senescent cells. **m** Boxplot showing the SASP score of senescent and not senescent cells (two sided Wilcox-test: *p* value < 2.2e−16; *N* = 3992 cells, Not senescent cells (left) minimum = −0.0537, lower-quartile = −0.0146, median = −0.0088, upper-quartile = −0.0039, maximum = 0.0408, Senescent cells (right) minimum = −0.0353, lower-quartile = 0.0018, median = 0.0154, upper-quartile = 0.0466, maximum = 0.1172). **n** Boxplot showing ss-gsva score of published gene set of Reactome collection^77^ (senescence_associated_secretory_phenotype) in senescent and not senescent cells (two sided Wilcox-test: *p* value < 2.2e−16; *N* = 3992 cells, Not senescent cells (left) minimum = 0.0634, lower-quartile = 0.1183, median = 0.1363, upper-quartile = 0.1607, maximum = 0.2952, Senescent cells (right) minimum = 0.0527, lower-quartile = 0.1527, median = 0.2183, upper-quartile = 0.2768, maximum = 0.4215). **o** Expression of fundamental SASP genes where dot size and color represent the percentage of cell expressing and the averaged scaled expression value, respectively. Source data are provided as a Source Data file.

To characterize senescent prostate cancer cells at single-cell resolution we collected the epithelial fraction of four prostate tumors from two different mouse models of prostate cancer, the Pten-null prostate conditional (*Pten*^*pc−/−*^*)* and the *Pten*^*pc−/−*^*; Timp1*^*−/−*^ mouse models^6,20^. These mice develop prostate tumors that evolve into locally invasive and metastatic prostate cancer, respectively. As previously reported, both *Pten*^*pc*−/−^ and the *Pten*^*pc*−/−^; *Timp*1^−/−^ prostate tumors are characterized by the presence of both a proliferative and a senescent compartment, as detected by positivity to SA-β-Galactosidase (SA-β-Gal) staining and the expression of different senescence markers (Fig. 1a, Supplementary Fig. 1a)^6,20^. Epithelial tumor cells (Epcam^+^) were FACS-sorted and analyzed by 10× Genomics single-cell RNA sequencing to obtain the transcriptomic profiles of almost 4000 cells (Fig. 1b, Supplementary Fig. 1b). By using a graph-based clustering method and Uniform Manifold Approximation and Projection (UMAP), we identified ten cancer cell clusters (Supplementary Fig. 2a–d). In order to identify senescent tumor cells at single-cells resolution, we defined a senescence signature by combining the expression of *p16*^*INK4a*^*, p15*^*INK4b*^*, p19*^*Arf*^*, p21*^*Waf1/Cip1*^*, p27*^*Kip1*^, and *PAI-1*, the most upregulated senescent markers in whole tumors lysates, as detected by western blot analysis^1,21^ (Fig. 1c, Supplementary Fig. 1a). Between the different identified clusters, clusters 3, 4, 5, and 7 showed the higher expression levels of this senescence signature (Supplementary Fig. 2e). Of note, while cluster 3, 4, 5 defined luminal prostate tumor cells, cluster 7 represented basal prostate tumor cells, thereby demonstrating that senescence can equally occur in cells of both compartments (Supplementary Fig. 2b). A major feature of senescent tumor cells is the absence of cellular proliferation, being these cells stably arrested^1,2,21,22^. By using different available gene sets (K, R, WP), through single-sample Gene Set Enrichment Analysis (GSEA) we further defined three cell cycle arrest signatures (Fig. 1d, Supplementary Fig. 2f). The combination of the senescence signature (Fig. 1c) with each of the three cell cycle arrest signatures allowed us to define three different Senescence Scores (SK, SR, SWP) (Fig. 1e, Supplementary Fig. 2g). We defined senescent cancer cells as the cells having the highest score level in SK, SR, and SWP (Senescence Index Tool, named SIT for short) (Fig. 1f). To validate the reliability of the SIT, we next investigated the differences between senescent and non-senescent prostate tumor cells at gene expression level. GSEA of the biological processes from Gene Ontology collection revealed selective activation and suppression of different pathways. Within the pathways with higher normalized-enrichment-score (NES), several were related to wound healing and migration (*locomotion*, *cell motility*, *epithelium migration*, *tissue migration*), all features characteristic of senescent cells^1,5,7^. On the contrary, oxidative phosphorylation and mitochondrial respiration were the most down-regulated pathways, in line with previous reports demonstrating that senescent cells have dysfunctional mitochondria and rely for their survival on the tricarboxylic acid cycle^23–25^ (Fig. 1g, Supplementary Data 1). Differential expression analysis between senescent and non-senescent prostate tumor cells showed that genes involved in transcription regulation, wound healing, SASP and oxidative phosphorylation were the most regulated (Fig. 1h, Supplementary Data 1). Among genes involved in transcriptional regulation, we found *c-Jun*, a pioneer transcription factor recently described as a master regulator of senescence, and additional genes encoding proteins that heterodimerize with *c-Jun* to form AP-1 complex, such as *c-Fos* and *Atf3*^26^ (Fig. 1i–k, Supplementary Fig. 2h). Accordingly, AP-1 downstream target genes were significantly upregulated in senescent cells (Supplementary Fig. 2i). Finally, we observed that senescent tumor cells overexpressed gene signatures involved in autophagy, NF-kB pathways activation, the SASP and previously validated senescence signatures, such as Fridman_senescence_up (Supplementary Fig. 2j). *RelA/p65*, a subunit of NF-kB and a key regulator of the SASP^27,28^, was the most upregulated gene of the NF-kB pathway, whereas *Cxcl1*, *Il6*, *Il1a* and *Cxcl15* were the most enriched SASP genes, in line with previous data from our and different research teams^5,27,28^ (Fig. 1l–o). Moreover, different out-of-clustering methods, such as scmap and SingleR^29,30^ using 12 different senescent datasets (from both available single cells and bulk RNA seq, Supplementary Table 1) validated the consistence of our tool (Supplementary Fig. 3a–f). Taken together, these data demonstrate that the SIT is a reliable tool to identify senescent tumor cells at single-cell level.

### Senescent tumor cells differently segregate at single-cell RNA-seq level based on their transcriptional profile

**Fig. 2:**
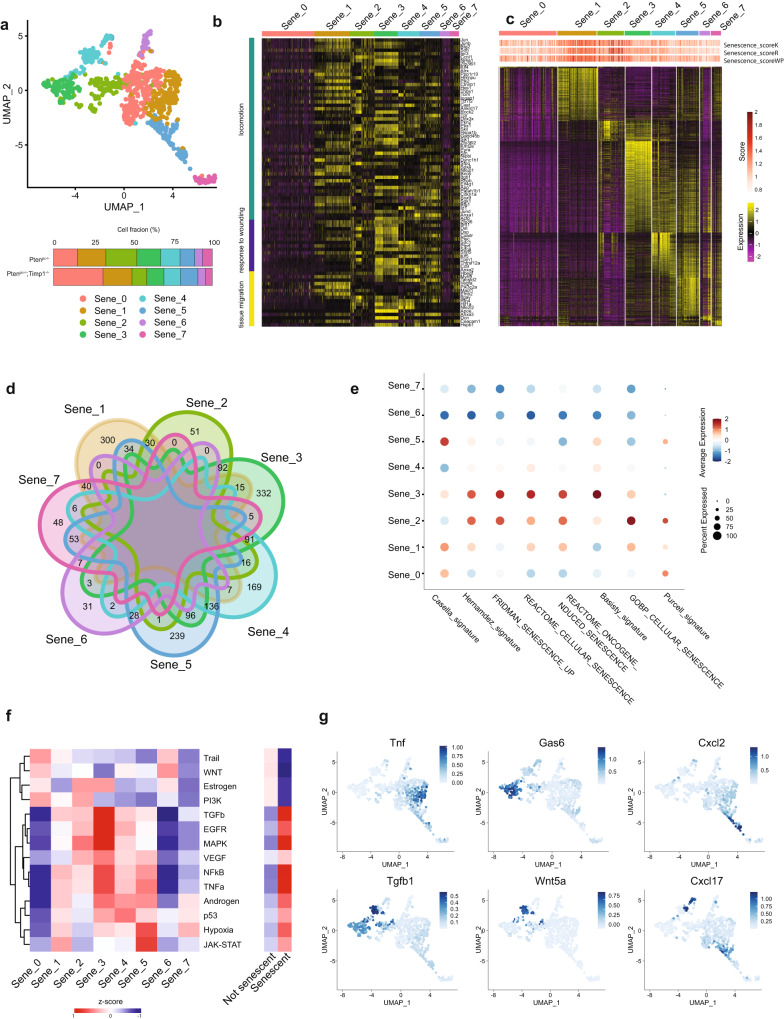
Senescent cells are a heterogeneous population. **a** UMAP view of only senescent cells, colored by clusters found with *FindCluster* function (res = 0.5) (top) and percentage of clusters in the two genotypes (bottom). **b** Heatmap showing expression of differentially expressed genes found in total senescent cells related to pathways activated in total senescent cells. **c** Heatmap showing differentially expressed genes between various senescent clusters (Sene_clusters) using *FindAllMarker* function. Wilcoxon Rank Sum test followed by FDR correction. **d** Venn diagrams showing the differentially expressed genes in each Sene_cluster. Overlapping areas indicate the number of genes commonly modulated among subpopulations. The numbers report only unique differentially expressed genes or transcripts common between two clusters. **e** Dotplot showing ssGSEA of previously published senescence signature (Casella_signature^31^, Hernandez_signature^32^, Fridman_senescence_up^33^, Basisty_signature^34^, Purcell_signature^35^, Cellular senescence from Gene Ontology collection, Oncogene-induced senescence and Cellular senescence gene sets from Reactome collection) in all the senescent clusters. **f** Heatmap showing pathway activity of 14 relevant signaling pathways calculated using PROGENy^80^ between different senescent subpopulations (left) and between senescent and not senescent cells (right). **g** FeaturePlot showing expression levels of different secreted factors upregulated in specific Sene_clusters and not expressed in the other senescent cells. Source data are provided as a Source Data file.

We then applied the SIT to explore the potential heterogeneity of senescent tumor cells and we found that these cells clustered in eight distinct cell subpopulations, regardless of the genetic background (Fig. 2a). Differential expression analysis of these eight subpopulations outlined commonalities and key differences. Sene_1, Sene_3 and Sene_5 were characterized by the over-expression of distinct gene expression programs compared to the other populations (Fig. 2b–d and Supplementary Data 2). Interestingly, the senescent clusters expressed different senescence signatures at different levels (Fig. 2e)^31–35^. Pathway analysis showed that JAK-STAT, NF-kB, p53, MAPK and TNFα signaling, known to be upregulated in senescent tumor cells, were mostly activated in the Sene_1–5 clusters. Instead, Sene_0 and Sene_6 clusters were characterized by the upregulation of WNT and PI3K patways^27,36–42^ (Fig. 2f). Interestingly, among the secretome of the different clusters, we found that *Tnf*, *Cxcl2*, *Cxcl17, Gas6*, *Wnt5a* and *Tgfb1* were specifically expressed by some of the Sene_clusters being undetectable in others (Fig. 2g). Previous data demonstrated that Cxcl2 and Cxcl17 are key chemokines involved in the recruitment of myeloid cells, which are the most enriched immune population in prostate cancers^43–45^. In sum, these data revealed that senescent tumor cells are heterogeneous at transcriptional level.

### Mcl-1 is the key factor that antagonizes cell death in senescent prostate tumor cells

**Fig. 3:**
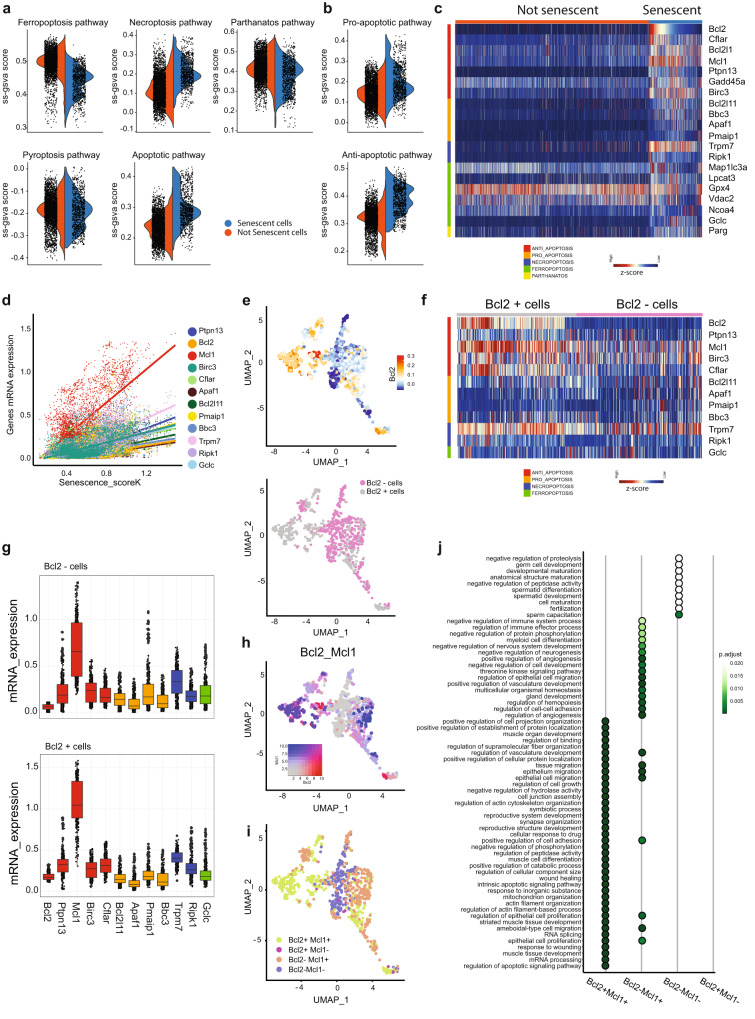
Survival mechanisms of senescent cells. **a** Violin plot showing ss-GSVA score activation of different cell death modalities between senescent and not senescent cells. **b** Violin plot showing ss-GSVA score activation of apoptosis between senescent and not senescent cells, separating in pro and anti-apoptotic genes. **c** Heatmap of 20 differentially expressed genes of different cell death modalities. FDR < 0.05. **d** Correlation plot between Senescence_scoreK and survival related-genes with Pearson correlation coefficient higher than 0.4 and FDR < 0.05. **e** UMAP plot showing Bcl2 expression levels in senescent cells (top) and classification of them in Bcl2+ or Bcl2- (bottom). **f** Heatmap showing *z*-score expression levels of 12 survival related-genes, positively correlated with Senescence_scoreK,WP,R, in Bcl2+ and Bcl2- senescent cells. **g** mRNA expression levels of 12 survival related-genes in Bcl2+ and Bcl2- senescent cells (*N* = 862 cells, Bcl2 - cells: Bcl2 median = 0.0596; Ptpn13 median = 0.1857; Mcl1 median = 0.654; Birc3 median = 0.234; Cflar median = 0.160; Bcl2l11 median = 0.140; Apaf1 median = 0.068; Pmaip1 median = 0.165; Bbc3 median = 0.091; Trpm7 median = 0.331; Ripk1 median = 0.169; Gclc median = 0.175. Bcl2 + cells: Bcl2 median = 0.169; Ptpn13 median = 0.324; Mcl1 median = 1.045; Birc3 median = 0.274; median = 0.314; Bcl2l11 median = 0.145; Apaf1 median = 0.083; median = 0.178; Bbc3 median = 0.113; Trpm7 median = 0.402; Ripk1 median = 0.261; Gclc median = 0.179). **h** Co-expression of Bcl2 and Mcl1 in senescent cells. **i** UMAP plot showing the classification of senescent cells based on Bcl2 and Mcl1 expression levels. **j** Dotplot showing over-representation results of biological process (GO) pathway analysis by considering senescent cell subclassification. Hypergeometric test followed by FDR correction. Source data are provided as a Source Data file.

We next focused on common genetic pathways expressed by these cells. Senescent tumor cells are known to be resistant to programmed cell death due to the upregulation of BCL2 and BCL-XL^1,2,46,47^. However, an extensive analysis of the pro-survival pathways upregulated in senescent prostate tumor cells has not been performed before. We therefore took advantage of the SIT to annotate pro-survival gene pathways deregulated in senescent prostate tumor cells in order to identify a common vulnerability (Fig. 3a). We found that senescent tumor cells upregulate pathways involved in necroptosis and apoptosis. By separating genes involved in pro-apoptotic and anti-apoptotic pathways, we found that the latter were significantly upregulated in senescent tumor cells (Fig. 3b, c). Among the identified pro-survival genes (*n* = 47) (Supplementary Fig. 4a), we found 12 genes that positively correlated (Pearson’s coefficient > 0.4) with the senescence scores (SK, SR, SWP). Among these, *Mcl-1*, a member of the BCL2 gene family, was the most correlated gene^48,49^ (Fig. 3d, Supplementary Fig. 4b). Of note, *Mcl-1* was more upregulated than *Bcl2*, a well-known target of senolytic therapy^50,51^ (Supplementary Fig. 4c). We next classified senescent tumor cells in two subpopulations based on *Bcl2* expression (Bcl2^+^ and Bcl2^−^) (Fig. 3e). Surprisingly, we found that roughly 50% of senescent tumor cells were not expressing *Bcl2* at high levels. On the contrary, *Mcl-1* was expressed both in the Bcl2^+^ and Bcl2^−^ clusters and it was the most upregulated gene in these clusters when compared to additional pro-survival genes (Fig. 3f–h, Supplementary Fig. 4d). Both Bcl2^+^Mcl1^+^ and Bcl2^−^Mcl1^+^ senescent cells overexpressed genes involved in the regulation of angiogenesis, migration and were enriched in SASP genes (Fig. 3i, j, Supplementary Fig. 4e–g, Supplementary Data 3). Altogether, these data suggest that the majority of senescent tumor cells rely on *Mcl-1* over-expression and that this cell population upregulates gene pathways that may contribute to tumor progression through different mechanisms.

### Inhibition of Mcl-1 works as a potent senolytic therapy

**Fig. 4:**
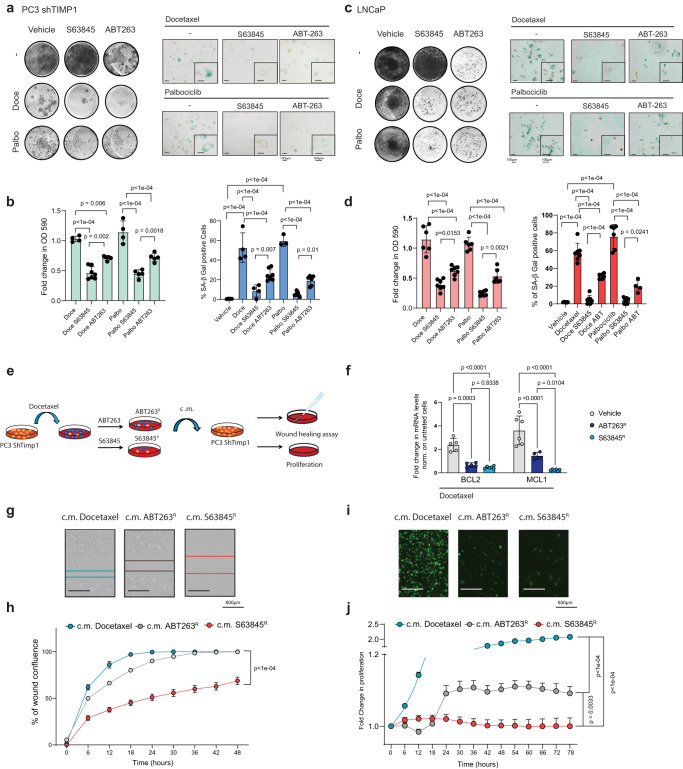
Mcl-1 targeting as senolytic anti-migratory therapy. **a** Representative pictures of crystal violet and SA-β Gal of PC3 shTIMP1 treated cells (Scale bar 100 μm). Data are representative of three independent experiments. **b** Cristal violet and SA-β Gal quantification (Cristal violet *n* = 4 for Docetaxel and Palbociclib, *n* = 5 for Docetaxel ABT263, Palbociclib S63845 and Palbociclib ABT263, *n* = 7 for Docetaxel S63846; SA-β Gal *n* = 4 for Vehicle, Docetaxel, Docetaxel S63845, *n* = 3 for Palbociclib, *n* = 6 for Palbociclib ABT263 and *n* = 7 for Docetaxel ABT263 and Palbociclib S63845, *n* = biological independent samples from three independent experiments). **c** Representative pictures of crystal violet and SA-β Gal of LNCaP treated cells (Scale bar 100 μm). Data are representative of three independent experiments. **d** Quantification of crystal violet and SA-β Gal staining (Cristal violet *n* = 6 for Docetaxel, Palbociclib and Palbociclib ABT263, *n* = 7 for Docetaxel S63845, Docetaxel ABT263 and Palbociclib S63845; *n* = biological independent samples, SA-β-Gal *n* = 6 for Vehicle, Docetaxel ABT263 and Palbociclib, *n* = 7 for Docetaxel, Docetaxel ABT263 and Palbociclib S63845, *n* = 4 for Palbociclib ABT263, *n* = biological independent samples from three independent experiments). **e** Schematic representation of the experimental design. **f** RT-qPCR analysis of Docetaxel treated cells and the remaining clones upon ABT263 (ABT263^R^) and S63845 (S63845^R^) treatment (for Bcl2 *n* = 5 for Vehicle and ABT263^R^, *n* = 6 for S63845^R^; for Mcl1 *n* = 6 for Vehicle and S63845^R^ and *n* = 4 for ABT263^R^, *n* = biological independent samples from three experiments). The *p* values were determined by one way ANOVA followed by Tukey’s multiple comparison test. **g** Representative pictures of wound healing assay (Scale bar 600 μm). Data are representative of two independent experiments. **h** Percentage of wound confluence over time normalized to time 0 (for c.m. Docetaxel and c.m. ABT263^R^ *n* = 8, for c.m. S63845^R^ *n* = 6, *n* = biological independent samples from two independent experiments). **i** Representative pictures of proliferation assay (Scale bar 600 μm). Data are representative of two independent experiments. **j** Fold change in proliferation normalized to time 0 (for c.m. Docetaxel, c.m. ABT263^R^ and c.m. S63845^R^ *n* = 6, *n* = biological independent samples from two independent experiments). **b**, **d**, **h**, **j** The *p* values were determined by one way ANOVA followed by Tukey’s multiple comparison test. The whole Anova results were given in the Supplementary Data 4. **b**, **d**, **f** Data are represented as mean ± SD. **h**, **j** Data are represented as mean ± SEM. Source data are provided as a  Source Data file.

Intrigued by these findings, we next checked whether *Mcl-1* was also overexpressed in cells treated with drugs that cause TIS. Human (PC3, PC3 shTIMP1 and LNCaP) and mouse (TrampC1, TC1, TrampC1^*Pten−/−*^, TC1^*Pten−/−*^, and RapidCap) prostate tumor cells were treated with Docetaxel and Palbociclib, in order to trigger a senescent response (Fig. 4a–d, Supplementary Fig. 5a, b and Supplementary Data 4). Establishment of senescence in these cells was associated to Mcl-1 and Bcl2 upregulation (Supplementary Fig. 5a, b). To assess whether *Mcl-1* contributed to the survival of senescent tumor cells, we treated them with S63845, a potent Mcl-1 inhibitor^52,53^ in parallel to the Bcl2 inhibitor Navitoclax (ABT263)^50,51^. Of note, we found that S63845 was capable to drive senolysis more efficiently than ABT263, both in terms of selectivity and potency, as visualized by SA-β-Gal staining and quantification of proliferation by crystal violet and Incucyte imaging analysis (Fig. 4a–d, Supplementary Fig. 5c). The removal of senescent cells by S63845 was accompanied by apoptosis as shown by upregulation of Cleaved Caspase 3 (Supplementary Fig. 5d). These data were further validated by using two additional inhibitors of Mcl-1, UMI77 and AZD5991^54,55^ (Supplementary Fig. 5e–h). Interestingly, after ABT263 treatment we found a population of ABT263 resistant (ABT263^R^) cells that was still SA-β-Gal positive, whereas S63845 treatment resulted in a smaller fraction of surviving senescent tumor cells (S63845^R^) (Fig. 4a, c). We next checked whether ABT263^R^ human cells also expressed Mcl-1. RT-qPCR analysis confirmed that ABT263^R^ cells upregulated Mcl-1 whereas S63845^R^ cells did not (Fig. 4e, f). We next used an inducible shMCL1 in LNCaP and RapidCap cells to validate Mcl-1 as senolytic target (Supplementary Fig. 5i). Doxycycline administration efficiently decreased the levels of Mcl-1 in both cell lines (Supplementary Fig. 5j, m). Of note, inactivation of Mcl-1 in cells treated with TIS efficiently eliminated senescent tumor cells (Supplementary Fig. 5k, l, n, o). In cells treated with Doxycycline and S63845 alone, the elimination of senescent cells was comparable (Supplementary Fig. 5l, o). However, in cells treated with Doxycycline and S6384 in combination, we did not find an increased percentage of dead cells (Supplementary Fig. 5l, o). These data demonstrate that senescent cells rely on Mcl-1 for their survival and that S63845 is a specific Mcl-1 inhibitor.

Thus, we took advantage of this model to assess whether senescent tumor cells resistant to senolytic therapy could impact on tumor cells proliferation and migration. In line with our previous findings^6^, while condition media (c.m.) from senescent PC3 shTIMP1 cells treated with Docetaxel increased the migration and proliferation of parental PC3 cells, c.m. from S63845 treated cells completely arrested the proliferation and migration of PC3 parental cells. This effect was superior to the one observed in cells treated with ABT263 (Fig. 4g–j). Intriguingly, co-culture experiments using Docetaxel treated GFP^+^-PC3 ShTIMP1 cells and untreated parental PC3 showed that a small fraction (15%) of senescent cells was capable to migrate. However, this was not observed in S63845 treated cells (Supplementary Fig. 6a–e). The effect on proliferation enhancement induced by c.m. derived from senescent cells was further validated in Tomato^+^-RapidCap cells treated either with c.m. or co-cultured with untreated parental cells. Also, in this case S63845 treatment was superior to ABT263 (Supplementary Fig. 6f–n). In sum, these data demonstrate that senescent tumor cells treated with TIS can migrate and induce the proliferation and migration of neighboring prostate tumor cells. These pro-tumorigenic effects can be efficiently abrogated by senolytic therapy with S63845.

### Mcl-1 inhibition enhances the efficacy of standard of therapy in prostate cancer

**Fig. 5:**
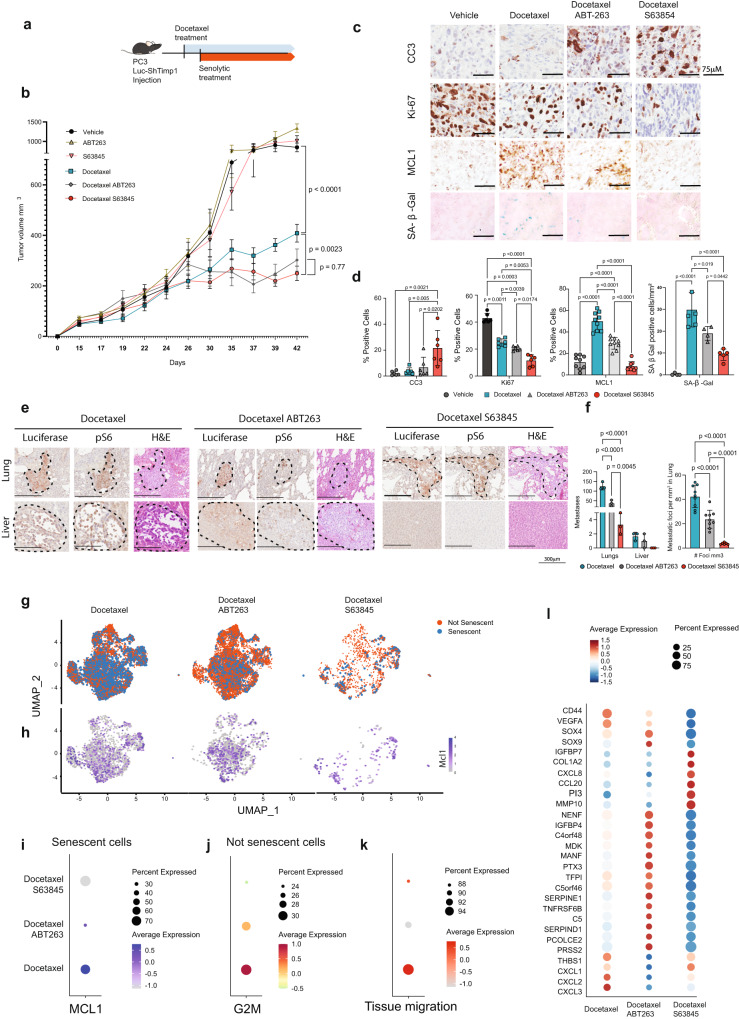
S63845 is an efficient senolytic therapy in vivo. **a** Schematic representation of the experimental design. **b** Growth curve of tumors in vivo in mm^3^ (*n* = 5 for Untreated, ABT263, S63845, Docetaxel, Docetaxel + ABT263, Docetaxel + S63845, *n* = independent animals from one experiment). The *p* values were determined by two-way ANOVA followed by Tukey’s multiple comparison test at time point 39 days post injection, separately for single treatment (Untreated, Docetaxel, ABT263, S63845) and Docetaxel treated groups (Docetaxel, Docetaxel + ABT263, Docetaxel + S63845). Data are represented as mean ± SEM. **c** Representative pictures of immunohistochemistry for Cleaved Caspase 3 (CC3), Ki67, MCL-1 and SA-β Gal. Data are representative of one experiment. **d** From left, quantification in percentage (CC3 *n* = 6, Ki67 *n* = 6, MCL-1 *n* = 9 and SA-β Gal *n* *=* 6; *n* = multiple areas of four animals from one experiment. The *p* values were determined by One-way ANOVA test followed by Tukey’s multiple comparisons test). Data are represented as mean ± SD. **e** H&E, Luciferase and phospho-S6 immunohistochemical staining in lung and liver metastases in Docetaxel, Docetaxel + ABT263 and Docetaxel + S63845 treated NRG mice. Data are representative of one experiment. **f** Bar graph representing metastases quantification (*n* = 9 for Docetaxel and Docetaxel ABT263, *n* = 6 for Docetaxel S63845; = multiple areas of 4 animals from one experiment). For metastases count the *p* values were determined by Two-way ANOVA followed by Šídák’s multiple comparisons, while for the count of metastases foci per mm^3^, the *p* values were determined by one Way ANOVA followed by Tukey’s multiple comparisons test. Data are represented as mean ± SD **g** UMAP plot of senescent and not senescent cells, defining using SIT, in xenograft PC3 shTIMP1 cells treated with Docetaxel alone or in combination with ABT263 or S63845 (Docetaxel % senescent cells = 35.5%, Docetaxel + ABT263 % senescent cells = 15.6%, Docetaxel + S63845 % senescent cells = 14%). **h** UMAP plot of senescent cells showing MCL-1 expression in xenograft PC3 shTIMP1 cells treated with Docetaxel alone or in combination with ABT263 or S63845. **i** MCL-1 expression for each treatment, where dot size and color represent the percentage of cells expressing the indicated gene and the average scaled expression value, respectively. **j** G2M signature for each treatment, where dot size and color represent percentage of cells expressing and the averaged scaled expression value, respectively. **k** ss-GSVA of tissue migration signature for each treatment, where dot size and color represent percentage of cell expressing and the averaged scaled expression value, respectively. **l** Marker genes expressions for each treatment, where dot size and color represent percentage of cell expressing and the average scaled expression value, respectively. Source data are provided as a Source Data file. The whole Anova results were given in the Supplementary Data 4.

To further corroborate these findings in vivo, we subcutaneously injected PC3 Luc-shTIMP1 in NRG mice. As previously shown, PC3 Luc-shTIMP1 tumor cells can migrate, invade the surrounding tissues and metastasize upon TIS due to the increased activity of MMPs^6^. When tumors reached the volume of 100 mm^3^, mice were treated with Docetaxel followed by either S63845 or ABT263 treatments (Fig. 5a, b). While Docetaxel increased senescence in primary prostate tumors, S63845 treatment promoted an efficient elimination of senescent prostate tumor cells, as assessed by SA-β-Gal and MCL-1 positive cells and the increased positivity for Cleaved Caspase 3 (Fig. 5c, d). Note that S63845 treatment was more effective than ABT263 in eliminating senescent tumor cells.

Importantly, while mice treated with Docetaxel developed metastases to the lungs and the liver, mice treated with the combination of Docetaxel and S63845 showed a stronger reduction in metastases formation. This effect was superior than in mice treated with Docetaxel in combination with ABT263 (Fig. 5e, f). 10× Genomics bioinformatic analysis using SIT in primary senescent prostate tumors cells showed that S63845 treatment resulted in a stronger elimination of senescent tumor cells when compared to ABT263 treatment (Fig. 5g, Supplementary Fig. 7a). Of note, in prostate tumor treated with Docetaxel and ABT263, the remaining senescent tumor cells upregulated MCL-1 and genes related to angiogenesis, cell migration and wound healing (Fig. 5h, i, Supplementary Fig. 7b). Moreover, these senescent tumor cells co-existed with a population of non-senescent cells that were more proliferative and pro-migratory than in tumors treated with Docetaxel and S63845 (Fig. 5j–l). This explains the reduction in Ki67 staining and the decreased number of metastases found in mice treated with S63845 (Fig. 5c–f). Elimination of senescent tumor cells and reduction in the expression levels of genes involved in metastases in mice treated with Docetaxel and S63845 was further validated by RT-qPCR in whole tumor lysates (Supplementary Fig. 7c, d). These data were further validated in *Pten*^*pc*−/−^ mice (Supplementary Fig. 8a). Mice treated with the combination of Docetaxel+ S63845 showed a marked reduction in the number of prostate glands affected by tumors when compared to mice treated with Docetaxel and Docetaxel + ABT263 (Supplementary Fig. 8b and Supplementary Fig. 9a). Next, we evaluated the efficiency of both S63845 and ABT263 in eliminating senescent cells in these tumors. Immunohistochemistry analysis for p16, SA-β-Gal and RT-qPCR for p21 and PAI-1 showed a reduction of senescent cells in mice treated with both S63845 and ABT263 (Supplementary Fig. 8c–e). However, S63845 had a stronger senolytic effect and promoted a stronger apoptotic response when compared to ABT263. Decrease in senescence markers was accompanied by MCL-1 and BCL2 reduction (Supplementary Fig. 8e). Treatment with S63845 and ABT263 also decreased the number of tumor infiltrating MDSCs and TAMs, the two most represented immune populations in prostate tumors characterized by loss of PTEN^46,56^ (Supplementary Fig. 8f, g and Supplementary Fig. 9b). Interestingly, we also detected a marked upregulation of Perforin, a marker of T and NK cells activation (Supplementary Fig. 8h).

## Discussion

The mechanism behind cellular senescence establishment, maintenance and survival remains an object of intense investigation^1,2^. Indeed, contrary to cell death, whose mechanisms of induction have been fully characterized, little is known about the mechanism by which tumor cells undergo senescence and remain alive in the tumor microenvironment. Recent data demonstrate that senescent tumor cells, which initially suppress tumor growth^7,57,58^, if not promptly removed by the tumor immune response, can promote the proliferation, migration and metastatization of bystander cells^1,4–6^. These effects have been ascribed to the SASP of the senescent tumor cells^1,4–6^. Indeed, although arrested, senescent cells can secrete in the tumor microenvironment a variety of factors that can stimulate non-senescent tumor cells to migrate or proliferate^6,10,12,21^. As recently shown, the genetic background of tumor cells can influence the SASP, that determines whether these cells act in tumor-suppressive or tumor-promoting processes^5,12,22,49,59^. By using single cell technology, we have now contributed with an additional piece of information to these previous findings, demonstrating that in tumors of the same genetic backgrounds, senescent tumor cells can be heterogeneous in terms of gene expression. This can pose challenges for the design of therapies that remove senescent tumor cells and for the future development of senolytics in clinical trials.

Most of the currently available senotherapies for cancers are still restricted to Bcl-2 targeting^2,3,50,51^. Here, we describe a population of senescent tumor cells that do not rely on Bcl-2 to survive. This population of cells upregulates Mcl-1 and after treatment with the Bcl-2 inhibitor Navitoclax, remains still capable to promote tumorigenesis through the SASP. Thus, regardless of senescence heterogeneity, our analysis identified Mcl-1 as a ubiquitous target to effectively remove senescent tumor cells. Mcl-1 is a BH3 protein that belongs to the BCL2 family, which controls apoptosis together with Bcl-2, Bcl2-L-10, Bcl-W and Bcl-xL. Mcl-1 has a different structure compared to the other members of the BCL2 family^60^. While ABT263 can efficiently block Bcl-2 and Bcl-xL, it cannot block Mcl-1^60^. On the other hand, second and third generation of Mcl-1 inhibitors are highly selective and nowadays they are used in some clinical trials^61^. We show that the efficacy of Docetaxel treatment, a standard of therapy for metastatic castration-resistant prostate cancer patients, can be enhanced by the concomitant administration of Mcl-1 inhibitors both in vitro and in vivo. Furthermore, treatment with different Mcl-1 inhibitors resulted in the effective removal of senescent tumor cells and the complete abrogation of the bystander migratory phenotype, orchestrated by the SASP on non-senescent tumor cells, both in transgenic and xenograft models. Moreover, this combination of compounds was superior in terms of efficacy to the combination of Docetaxel with ABT263. Of note, in the transgenic mouse model, elimination of senescent tumor cells was associated to reactivation of the tumor immune response as demonstrated by the decreased infiltration of MDSCs and upregulation of perforin, a marker of T cells activation.

In sum, senescent cells are highly heterogenous, but ultimately rely on a common pro-survival factor, Mcl-1. Importantly, this study endorses Mcl-1 inhibitors as a class of highly effective senolytics. Interestingly, a previous study on breast cancer showed that the senolytic sensitivity of ABT263 is controlled by NOXA, an inhibitor of Mcl-1^62^. While senescent cells with a high level of NOXA respond to ABT263, cells with a low level are resistant to ABT263 and respond to Mcl-1 inhibitor thereby validating our results in a different system^62^. Finally, senescent tumor cells share common features with aged cells. Thus, if validated in other models, these findings could be relevant also to ameliorate aging and age-related pathologies.

## Methods

### Mouse models

All mice were maintained under specific pathogen-free conditions in the animal facilities of the IRB institute. Experiments were performed according to the state guidelines and approved by the local ethical committee (“Dipartimento della Sanità e Socialità, Esperimenti su animali”, Canton Ticino), authorization number TI-51/2018 (Maximum tumor volume authorized = 1500 mm^3^, not exceeded). Prostate-specific *Pten*^*pc*−/−^ transgenic mice^20^ were crossed with Timp1^−/−^ mice (Jackson Laboratory, 6243) to generate Timp1 knock out in *Pten*^*pc*−/−^^6^. NRG male mice, at 12 weeks of age, were used for subcutaneous cell injection of PC3 shCtrl and shTIMP1. Prostate-specific *Pten*^*pc*−/−^ transgenic male mice at 12 weeks of age were used for in vivo experiments. Mice used for 10× scRNA sequencing were euthanized at 10 weeks of age. NRG mice were  challenged with PC3 shTIMP1 at 8 weeks of age and then monitored and kept under treatment up to 42 days post-injection. Finally, Pten^*pc*−/−^ mice were treated at 10 weeks of age and euthanized upon 6 weeks of treatment.

### In vivo treatments

Docetaxel (TEVA Pharma AG 6984894) intraperitoneally at 10 mg/kg once a week. ABT263 (MedChemExpress HY-10087) by oral gavage 50 mg/kg, daily. S63845 (MedchemExpress HY-100741) at the dose of 25 mg/kg by oral gavage. Mice were monitored for any suffering of distress or weight loss by measuring weekly total body weight of mice and monitoring the behavioral changes every day for a total of 4 weeks of treatment.

### Prostate cancer cell culture

PC3 and LNCaP human prostate cancer cells were purchased from ATCC (Cat. N CRL-1435^™^ and CRL-1740^™^ respectively) and were cultured according to the manufacturer’s instructions. Cells were cultured in RPMI 1640 supplemented with 10% FBS and 1% P/S. HEK-293T (human embryonic kidneys, Cat. N CRL-3216^™^) and TrampC1 (Cat. N CRL-2730^™^) cells were obtained from ATCC. RapidCap were obtained from Trottman laboratory^63^. Cells were cultured in DMEM supplemented with 10% FBS and 1% P/S. All cell lines were kept under controlled temperature (37 °C) and CO_2_ (5%) and used for experiments at early passages. All the cell lines were tested negative for Mycoplasma (MycoAlertTM mycoplasma detection kit, LT07-418, LONZA).

### In vitro treatments

Palbociclib was used at the concentration of 10 μM; Docetaxel was used at 1 nM; ABT263 was used at the dosage of 2.5 μM; S63845 was used at 10 μM; UMI-77 was used at 10 μM; AZD5991 was used at 10 μM.

### Generation of shTIMP1 human prostate cancer cell line

PC3 cell line was transfected with shRNA using the human TIMP1-directed shRNA RHS4430-200284918-V3LHS_317110. To prepare lentiviral particles, HEK-293T cells were transfected using JetPRIME transfection reagents (JetPRIME, Polyplus transfection, 114-07/712-60) as per the manufacturer’s instructions. PC3 cells were infected with the filtered lentiviral supernatant obtained from transfected HEK-293T cells. Infected human prostate cancer cells were subsequently selected using Puromycin (3 mg/ml).

### Generation of Pten^−/−^ TrampC1 murine prostate cancer cell line

TrampC1 cells were purchased from ATCC and cultured according to manufacturer’s instructions (DMEM, 10% heat-inactivated FBS, 100 U/ml penicillin, 0.1 mg/ml streptomycin). The transfection of the PTEN CRISPR/Cas9 KO plasmid (Santa Cruz Biotechnology sc-422475) was performed using jetPRIME^®^ transfection reagent according to the manufactory protocol at the ratio of 1:2 DNA/jetPRIME^®^. 24 h after transfection, the GFP transduced cells were sorted to purity 99% and plated as single cell on 96-well plates. At day 7 after cell sorting the resulting cell colonies were moved into 24-well plates for further expansion.

### Generation of shMCL-1 human and murine prostate cancer cell lines

LNCaP and RapidCap cell lines were transfected with shRNA using the human and mouse MCL-1-directed shRNA RHS4696-200751526 and RMM4431-200332978, respectively. To prepare lentiviral particles, HEK-293T cells were transfected using JetPRIME transfection reagents (JetPRIME, Polyplus transfection, 114-07/712-60) as per the manufacturer’s instructions. Both LNCaP and RapidCap cells were infected with the filtered lentiviral supernatant obtained from transfected HEK-293T cells. Infected prostate cancer cells were subsequently selected using Puromycin (3 mg/ml). The shRNA was activated in both cell lines adding Doxycycline in the cell culture media (1 mg/ml).

### Immunohistochemistry (IHC)

IHC tissue sections were processed as follows: deparaffinizaction, unmasking, pre-staining, blockings and secondary stainings. Deparaffinization was performed using three-step procedure. In the first and second step, the slides were immersed in OTTIX plus solution (Diapath, Cat No. X0076) for 5 min each followed by third and last step of OTTIX shaper solution (Diapath, Cat No. X0096) for 5 min. The slides were drained off the excess solution and were then immersed in ionized water for 5 min. Further, un- masking or antigen retrieval procedure was followed which involved immersing the section slides in pH solutions (depending upon the antibodies) at either pH 6 (Citrate, Company: Diapath, Cat No. T0050) or pH 9 (DAKO, Cat No. K800421-2) in water bath at 98 °C for 20–25 min. The slides were allowed to cool at room temperature for 20–25 min. The section slides were washed with 1xPBST (0.5% Tween20), two times for 3 min each, followed by staining procedure. Blocking procedure began by incubating the slides with 3% H_2_O_2_ (VWR chemicals, Cat no: 23615.248) for 10 min followed by 1xPBST washes as before and performing protein block. Protein blocking was performed using Protein-Block solution (DAKO Agilent technologies, Cat No. X0909) for 10 min at room temperature. Depending upon antibodies, if they were developed in mouse host, the tissues were blocked for mouse cross-reactivity using biotinylated Anti-Mouse antibody (Vector Laboratories, Cat No. BP-9200). Sections were stained with respective primary antibodies at room temperature for 1 h followed by three washes with 1xPBST as before. These slides were further incubated with respective secondary antibodies, Anti-Mouse (Vector Laboratories, Cat No. BP-9200), Anti-Rabbit (Vector Laboratories, Cat No. BP-9100). During secondary antibody incubation, Vectastain ABC solution was prepared (Company: Vector laboratories, Cat No. PK-6100) at the dilution of 1:150 of both Solution A and Solution B in 1xPBS solution followed by 30 min incubation at room temperature. Upon completion of secondary antibody stainings, slides were washed for three times with 1xPBST followed by ABC solution staining for 30 min at room temperature. After ABC, slides were washed three times with 1xPBST and final steps of IHC stainings were performed. DAB staining was performed using DAB solution (Company: Vector laboratories, Cat No. SK-4105. One drop of Chromogen in 1 ml of Diluent solution) and allowed to stain for no more than 3–4 min at room temperature. Immediately slides were washed three times with 1xPBST and counter staining was performed using hematoxylin solution (Diapath, C0303). At the end of IHC staining, sections were dehydrated using deparaffinization procedure after which slides were mounted with coverslip using aqueous mounting media (Diapath, 060200). Tumor tissue samples were fixed in 10% neutral-buffered formalin (Thermo Scientific, Cat No. 5701) overnight. Tissues were washed thoroughly under running tap water followed by processing using ethanol and embedded in paraffin according to standard protocols. Sections (5 mm) were prepared for antibody detection and hematoxylin and eosin staining. Images were scanned with Aperio and opened with ImageScope v12.3.2.8013 (Leica Biosystem).

### Senescence associated β-galactosidase (SA-β-gal) assay

For tissue-specific SA-β-gal assay, tumor samples were immediately frozen in OCT solution at −80 °C and sections of 4 mm were prepared. Senescence-associated SA- β -gal staining was performed using Senescence β -Galactosidase Staining Kit (Cell Signaling Cat. No 9860) according to the manufacturer’s instructions. Counter staining was performed using Eosin staining (Alcohol-based Diapath, C0352). For in vitro experiment, SA- β -gal staining was performed using Senescence β -Galactosidase Staining Kit (Cell Signaling Technology, Cat. No 9860) according to the manufacturer’s instructions.

### Proliferation and cell death assay

Proliferation assay in PC3, TrampC1, Pten^−/−^ TrampC1 and Rapidcap cell lines was performed by plating 1 × 10^4^ cells per well of a 96-well plate in at least sextuplicate. Proliferation was monitored and analyzed by using Incucyte S3 in vitro system (Essenbioscience).

### Migration assay

Migration assay was performed in PC3 by plating 20 × 10^4^ cells per well of 96-well plate in at least sextuplicate. Wound was performed using Incucyte Wound Maker and cell migration was monitored and analyzed by using Incucyte S3 in vitro system (Essenbioscience).

### Condition media assay

Cell supernatants were harvested and spun down at 453 g for 10 min and the supernatant was filtered using 0.22 mm filters. Conditioned medium was administered to parental cells at time zero or 48 h prior the assay for proliferation and migration assay, respectively. The conditioned medium in all the experiments was normalized based on the number of cells present in the well at the moment of the harvesting.

### Western blot

Prostate tissues, tumor samples or cells were lysed using 1x RIPA buffer (Cell signaling, 9806) supplemented with Phenylmethanesulfonyl fluoride (PMSF; Millipore Sigma, catalog 329-98-6) and incubated on ice for 30 min. Samples were centrifuged at 46357 *g* for 15 min. Protein concentration was determined by the BCA kit (Thermo Fisher 23227). Equal amounts of proteins were subjected to SDS-polyacrylamide gel electrophoresis (SDS-PAGE), 10% and transferred on to 0.45 mm nitrocellulose membrane (Thermo Scientific, 88018). After protein transfer, membranes were blocked in 5% milk solution and membranes were probed with the indicated antibodies overnight at 4 °C. The membranes were incubated with horseradish peroxidase-conjugated (HRP-linked) secondary antibodies anti-rabbit IgG (Promega, W4011, 1:5000) or anti-mouse IgG (Cell signaling, W4021, 1:5000) and developed using enhanced chemoluminescence (ECL) substrate (Thermo Scientific, 32106). Membranes were exposed to Fusion Solo S imaging system (Vilber). Blots were semi-quantitatively analyzed by densitometry using ImageJ 1.52 v (National Institutes of Health).

### Antibodies

For IHC anti-Cleaved Caspase 3 (Cell signalling #9661), anti-Ki67 (RTU-Lab Vision #RM-9106-R7 Dilution Ready to use), anti-Luciferase (Abcam #ab181640), Mcl-1 (Cell signaling #5453) Ly-6G (GR1), Clone 1A8 (RUO); 551459 BD Pharmigen, F4/80 (BM8) Rat Mono, 14-4801-82 eBioscience^™^ (Thermo Scientific), p16 (Abcam #ab211542) were used. For Western blot anti-Cleaved Caspase 3 (Cell signalling #9664), anti-Bcl-2 (Cell signalling #3498 S), anti-Mcl-1 (Cell signalling #5453) anti-HSP90 (Cell signalling #4874), p27 kip1 (Cell signalling #3698 S), p15 ink4b (Abcam #53034), p16 ink4a (Abcam #211542), p21 (Abcam #107099), p19 ARF (5-C3-1) (Santa Cruz Biotechnology #SC-32748), PAI-1 (Abcam #66705), Mcl-1 (Cell signaling #5453), secondary Anti-Rabbit (Promega #W4011), secondary Anti-Mouse (Promega #W4021). For FACS sorting CD326 (EpCAM) Monoclonal Antibody (G8.8), FITC, eBioscience^™^ (11-5791-82). Further information about dilutions and clones are available in Source Data.

### Quantitative real-time PCR (RT-qPCR)

RNA extraction from cells or tissues samples was performed using Trizol (Ambion, life technologies, 15596026), according to the manufacturer’s instructions. cDNA was obtained using ImPROM II kit (Promega, A3800) according to the manufacturer’s instructions. RT-qPCR was performed using Gotaq^®^ qPCR Master Mix, Promega^®^ (A6002) on Step One Real-Time PCR systems (Applied Biosystems). Primers used for RT-qPCR are listed in Supplementary Table 2. Expression levels were calculated using the ddCT method^64^.

### Single cell sequencing analysis

Prostate tumors were resected from Pten^*pc*−/−^ and Pten^*pc*−/−^; Timp1^−/−^ mice (all three lobes, AP, DLP and VP) and from NRG mice injected subcutaneously with 2.5 * 10^6^ PC3 ShTIMP1 (see “Data availability” section). Sample were processed for single cell suspension followed by RNA sequencing and analysis using the following procedures:

### Single cell suspension

Prostate tumors or xenografts tumors were isolated, minced and processed for single cell suspension. Tissues were digested in 2 ml of Digestion Buffer composed by RPMI 10% FBS + 1% P/S, 500 mL of Collagenase D (1 mg/mL), 50 mL of DNAse (100 U/mL) and 125 mL of HEPES (25 mM). The cell suspension was incubated for 50 min at 37 °C on a rocker. Then, the digestion was stopped by adding 1 mL of RPMI 10% FBS + 1% P/S. The cells suspension was filtered through a 100 μm cell strainer and kept on ice for 4 min. Then cells suspension was filtered again through a 40 μm cell strainer and spun down at 453 g for 5 min at 4 °C. FACS staining was performed using EPCAM-FITC (anti-Mo CD326, eBioscience, clone G8.8 #11-5791-82) for the murine prostate tumors, while PC3 shTIMP1 were sorted thanks to GFP positivity. Samples were acquired on a BD sorter Aria III (BD Biosciences). The software used was BD FaCSDiva v9.0. No further analyses were needed for this study. The gating strategy used in transgenic mouse models have been done as it follows: FSC-H/FSC-A, SSC-A/FSC-A, 7AAD/FSC-A, EPCAM + /FSC-A (Supplementary Fig. 1). While in xenograft models FSC-H/FSC-A, SSC-A/FSC-A, GFP + /FSC-A (Supplementary Fig. 6a).

Single-cell transcriptomes was performed using 10× Chromium single cell platform (10× Genomics, Pleasanton, CA. USA). FACS-sorted Epcam+ prostate cells were used as the input source for the scRNA-seq. Cells were suspended in a phosphate buffer solution containing 0.04% weight/volume bovine serum albumin (BSA). The recommended volume of single cell suspension was loaded on a Chromium Single Cell Controller (10× Genomics) targeting ~10,000 cells per murine prostate samples while for xenografts tumors we used a target cell recovery between 5000 and 10000 cells per sample. Bar coded single-cell gel beads in emulsion (GEMs) were created by 10× Genomics! Chromium TM and then reverse transcribed to generate single-cell RNA-seq libraries using Chromium Single Cell 3′ Library and Gel Bead Kit v2 (10× Genomics) according to manufacturer’s instructions. Resulting short fragment libraries were checked for quality and quantity using an Agilent 2100 Bioanalyzer and Invitrogen Qubit Fluorometer. Unique molecular identifiers (UMIs), which were incorporated into the 5′ end of cDNA during reverse transcription, were used to quantify the exact number of transcripts in a cell.

### Single-cell sequencing data preprocessing and quality control

Sequencing data were processed by CellRanger^65^ (version 3.1.0 for murine alignment and version 6.0.0 for xenograft derived-samples) and reads were aligned to mouse genome (mm10 v3.0.0) or human genome (GRCh38) with STAR^66^ (v.2.5.1b). To reduce the ‘dropout’ phenomenon, RMagic package was used on gene-counts^67^. Single cell sequencing analysis was performed in epithelial cells purified from both Pten^*pc*−/−^ and Pten^*pc*−/−^; Timp1^−/−^ tumors or on GFP^+^ cells in PC3 shTIMP1 xenograft. For each cell, we calculated different quality measures: percentage of mitochondrial genes, number of genes and gene biotype. We removed cells that had more than 25% expression on mitochondrial genes, fewer than 100 total genes expressed and we considered only protein coding genes defined using *getBM* (biomaRt package). All the samples were integrated using Seurat^68–71^ package (*FindIntegrationAnchors* and *IntegrateData* function after library-size normalization of each cell using *NormalizeData* function with default parameters).

We identify features that are variable in the samples with *FindVariableFeatures* function and Principal component analysis (PCA) dimensionality reduction was run using the top 2000 features identified. Number of the included components (PCs) was assessed using the *ElbowPlot* function and fifteen PCs were conserved. Graph-based clustering approach was used to cluster the cells using *FindNeighbours* (*k* = 20) and *FindClusters* functions (0.25 < res < 2 with 0.25 difference). To visualize the data, the dimensional reduction technique t-distributed stochastic neighbor embedding (t-SNE) and UMAP^72^ were applied using the *RunTSNE* and *RunUMAP* functions from Seurat.

In murine single cell sequencing, cells positive for CD45 (Ptprc gene) and negative for Epcam were removed. Inside the Epcam+ cells, we defined epithelial subtypes based on canonical markers: Cd24a, Krt8 and Krt18 for luminal cells; Trp63, Krt5 and Krt14 for basal cells and Pax2, Pate4 and Calml3 for semi-vesical cells^73^. For xenograft sc-RNAseq data, all the analysis were performed after regression of nCount_RNA and nFeature_RNA features, due to differences between the samples.

### Senescence index tool (SIT)

To define senescent cells, we developed an algorithm based on common features of senescent cells: overexpression of specific genes and cell cycle arrest. The tool was composed of four steps: (a) definition of the senescence signature; (b) definition of cell cycle arrest signatures; (c) co-occurence of (a) and (b) defined by senescence scores K, WP and R; (d) identification of senescent cells based on senescence score K, WP and R.

#### Definition of senescence signature

AddModuleScore function (Seurat package) to define the average expression of known senescent markers (p16, p15 p19, p21, p27 and PAI-1) in each cell.

#### Definition of cell cycle arrest signatures

ss-GSVA score was calculated for each cell with GSVA package by using three different gene sets (KEGG CELL CYCLE^74–76^; REACTOME CELL CYCLE and WP CELL CYCLE^78^). To define the cell cycle arrest we inverted the direction of scores:

$$\left(1\right)\,{Cell}\,{Cycle}\,{arrest}\,{signatur}{e}_{i}=-{ssGSVA}\,{scor}{e}_{i}\,{with}\,(i=K,{WP},R)$$

#### Definition of senescence scores K, WP and R

We combined the different cell cycle arrest signatures with the senescence signatures by using these formula:

$$\left(2\right)\, {Senescence}\,{scor}{e}_{i}={Cell}\,{cycle}\,{arrest}\,{signatur}{e}_{i\,{norm}\left(0-1\right)}+{Senescence}\,{signatur}{e}_{{norm}\left(0-1\right)}$$

$${with}\,(i=K,{WP},R)$$

Identification of senescent cells: we divided the senescence score K, WP and R based on quantile division (quantile function) and we considered as senescent cells the one in which the senescence score K, WP and R were belonged to the fourth quartile (highest value).

### Single-cell sequencing data processing

Differentially expressed genes were identified using *FindAllMarkers* or *FindMarker* functions. Genes were identified as differentially expressed in a particular set of cells if FDR < 0.05 and minimum expression in at least 30% of cells.

Pathway analysis were performed using clusterProfiler^79^ package: for enrichment analysis we used *gseGO* (ont = “BP”) function while for over-representation analysis we used *enrichGO* function. The outputs of pathway analysis were simplified using simplify function with cutoff = 0.6 and gene sets were considered significant with FDR < 0.05. In case of multiple comparisons of pathway, we used *compareClusterResult* function. To analyze the activity of 14 relevant signaling pathways, we used Pathway RespOnsive GENes (PROGENy^80^) analysis. To study senescence heterogeneity, all the analyses were performed using exclusively the genes found upregulated in total senescent cells compared to non senescent cells (3757 genes).

ss-GSVA scores for different published gene sets and cell death pathways were calculated using gsva^81^ function (method = “ssgsea”). Correlation analysis was performed using cor.test function (alternative = two.sided, method = “pearson”, conf.level = 0.95) and adjusted *p* values were calculated with p.adjust function using (method = “BH”). For classification of cells based on Mcl1 expression levels we classified into quartiles: Mcl1+ corresponds to first, second and third quartiles, while for Bcl2 expression we used the median levels to classify in Bcl2+ and Bcl2-. All plots were designed using Seurat package and ggplot2 package.

For out-of-clustering methods, cells were projected to the combination of 12 senescence-related available datasets (Supplementary Table 1, see “Data availability” section) using scmap-cluster v1.16.0 (threshold = 0.15) and the function SingleR from SingleR package with default parameters.

### Signatures

Apoptotic pathway: Bcl2, Bcl2l1, Mcl1, Bcl2l12, Bcl2a1a, Cflar, Gadd45a, Traf1, Ptpn13, Birc3 (anti- apoptosis), Bbc3, Bcl2l11, Pmaip1, Bak1, Bax, Pidd1, Bid, Apaf1, Fas, Tnfrsf10b (pro-apoptosis); Ferroptosis pathway: Map1lc3a, Atg5, Atg7, Ncoa4, Alox15, Lpcat3, Acsl4, Vdac2, Vdac3, Cybb, Gpx4, Gss, Gclc; Necroptosis pathway: Mlkl, Trpm7, Ripk1, Ripk3; Parthanatos pathway: Aifm1, Rnf146, Parp1, Parg; Pyroptosis pathway: Gsdmd, Nlrp3, Nlrc4, Aim2,

Casp1, Pycard, Mefv; Sasp signature: Cxcl1, Cxcl2, Hc, Csf3, Csf2, Csf1, Il10, Il13, Il6, Cxcl13, Cxcl10, Icam1, Ccl2, Cxcl15, Ccl20, Vegfc, Vegfa, Inhba, Il1a, Il1b, Bmp2, Gdf15, Tgfb1, Tgfb2, Tgfb3, Bmp6, Ogg1^45,82^.

### Quantification and statistical analysis

All experiments were performed on biological replicates as mentioned in the respective figure legends. Sample size for each experimental group/condition is reported in the appropriate figure legend. All data points are presented for quantitative data, with an overlay of the mean with SEM. Statistically significant differences between control and experimental groups were determined using Multiple Student’s *t* tests (two-tailed, unpaired), one way ANOVA with Tukey multiple comparison difference test, Wilcoxon test, and log-rank (Mantel–Cox) test as indicated in the appropriate figure legend and text. All statistical analyses were performed using GraphPad Prism 8, Microsoft Excel 2016 or R-Studio.

### Reporting summary

Further information on research design is available in the Nature Research Reporting Summary linked to this article.

## Supplementary information

Supplementary Information

Description of Additional Supplementary Files

Supplementary Data 1

Supplementary Data 2

Supplementary Data 3

Supplementary Data 4

Supplementary Data 5

Reporting Summary

## Data Availability

The single-cell RNA sequencing data generated in this study have been deposited in the Gene Expression Omnibus (GEO) database under accession code GSE189519 for xenograft prostate cancer model and GSE189307 for murine prostate cancer models. The publicly available RNA-seq data used in this study are available in GEO (Gene Expression Omnibus), and EMBL-EBI databases under accession codes GSE115301^83^, GSE61130^84^, GSE130727^31^, GSE102639^85^, GSE132369^86^, GSE98440^87^, GSE109270^88^, GSE158743^89^ and E-MTAB-9970^90^. The source data of the figures are provided as Source Data files. Source data are provided with this paper.

## Source data

Source Data

## Code availability

The code for Senescence Index Tool (SIT) is available as Supplementary Data 5. All the other analyses are done with standard pipelines and are fully described in the Methods section. Source data are provided with this paper.
