# Author Correction: Single-cell transcriptomics identifies Mcl-1 as a target for senolytic therapy in cancer

**DOI:** 10.1038/s41467-023-40080-9

**Published:** 2023-07-20

**Authors:** Martina Troiani, Manuel Colucci, Mariantonietta D’Ambrosio, Ilaria Guccini, Emiliano Pasquini, Angelica Varesi, Aurora Valdata, Simone Mosole, Ajinkya Revandkar, Giuseppe Attanasio, Andrea Rinaldi, Anna Rinaldi, Marco Bolis, Pietro Cippà, Andrea Alimonti

**Affiliations:** 1 Institute of Oncology Research (IOR), Oncology Institute of Southern Switzerland (IOSI), CH6500 Bellinzona, Switzerland; 2 Università della Svizzera Italiana, CH6900 Lugano, Switzerland; 3 Bioinformatics Core Unit, Swiss Institute of Bioinformatics, TI, 6500 Bellinzona, Switzerland; 4 Faculty of Biology and Medicine, University of Lausanne UNIL, CH1011 Lausanne, Switzerland; 5 Institute of Molecular Health Sciences, ETH Zurich, CH8093 Zurich, Switzerland; 6 Massachusetts General Hospital Cancer Center, Harvard Medical School, Charlestown, MA 02129 USA; 7 Department of Medicine, Division of Nephrology, Ente Ospedaliero Cantonale, Lugano, Switzerland; 8 Laboratories for Translational Research, Ente Ospedaliero Cantonale, Bellinzona, Switzerland; 9 Computational Oncology Unit, Department of Oncology, Istituto di Ricerche Farmacologiche ‘Mario Negri’ IRCCS, 20156 Milano, Italy; 10 Department of Health Sciences and Technology (D-HEST) ETH Zurich, 8093 Zurich, CH Switzerland; 11 Department of Medicine & Veneto Institute of Molecular Medicine, University of Padova, Padova, Italy

**Keywords:** Bioinformatics, Cancer models, Senescence, Cancer genomics, Targeted therapies

Correction to: *Nature Communications* 10.1038/s41467-022-29824-1, published online 21 April 2022

The original version of this Article contained errors in Fig. 4C and in the Supplementary Information file.

In Fig. 4C, the lower magnification image for the LNCaP Palbo + ABT263 image was a duplication of the LNCaP Docetaxel image.

In Supplementary Fig. 8C, the high magnification image obscured the region of interest.

In Supplementary Fig. 8F, the immunohistochemistry image for Docetaxel-treated Pten-/- MDSCs was a duplication of the macrophage image.

The figure legend of Supplementary Fig. 9b incorrectly read ‘Zoom out of pictures related to immune subset infiltrating the prostate tumor in Supplementary Fig. 6g (Scale bar 300 μm). Dashed squares represent areas visualized in Supplementary Fig. 6g.’ The correct version states ‘Supplementary Fig. 8f’ in place of ‘Supplementary Fig. 6g’.

These errors have been corrected in the PDF and HTML versions of the article. The HTML has been updated to include a corrected version of the Supplementary Information.

A pdf file containing the raw data for Fig. 4C, Supplementary Fig. 8C, and Supplementary Fig. 8F is appended below.

## Supplementary information

Updated Supplementary Information file

Merged Replicate Data

