# Supplementary material for: Single-cell transcriptomics identifies Mcl-1 as a target for senolytic therapy in cancer
Source: Nat Commun. 2022 Apr 21;13:2177. doi: 10.1038/s41467-022-29824-1 (PMC9023465; doi:10.1038/s41467-022-29824-1)
Supplement: Supplementary file 2 — Description of Additional Supplementary Files [file 41467_2022_29824_MOESM2_ESM.pdf]

Description of Additional Supplementary Files

File Name: Supplementary Data 1
Description: Pathway analysis and differential expression analysis results of senescent cells compared to not senescent cells.

File Name: Supplementary Data 2
Description: Differential expression analysis results of different senescent clusters.

File Name: Supplementary Data 3
Description: Pathway analysis of senescent cells classified based on Bcl2 and Mcl1 expression.

File Name: Supplementary Data 4
Description: Statistic results for main and supplementary figures.

File Name: Supplementary Data 5
Description: R code for Senescence Index tool (SIT).
