# Supplementary material for: Single-cell transcriptomics identifies Mcl-1 as a target for senolytic therapy in cancer
Source: Nat Commun. 2022 Apr 21;13:2177. doi: 10.1038/s41467-022-29824-1 (PMC9023465; doi:10.1038/s41467-022-29824-1)
Supplement: Supplementary file 8 — Reporting Summary [file 41467_2022_29824_MOESM8_ESM.pdf]

nature portfolio

Corresponding author(s): Andrea Alimonti

Last updated by author(s): Feb 21, 2022

Reporting Summary

Nature Portfolio wishes to improve the reproducibility of the work that we publish. This form provides structure for consistency and transparency in reporting. For further information on Nature Portfolio policies, see our Editorial Policies and the Editorial Policy Checklist.

Statistics

For all statistical analyses, confirm that the following items are present in the figure legend, table legend, main text, or Methods section.

n/a	Confirmed	
☐	☒	The exact sample size (n) for each experimental group/condition, given as a discrete number and unit of measurement
☒	☐	A statement on whether measurements were taken from distinct samples or whether the same sample was measured repeatedly
☐	☒	The statistical test(s) used AND whether they are one- or two-sided<br>*Only common tests should be described solely by name; describe more complex techniques in the Methods section.*
☒	☐	A description of all covariates tested
☐	☒	A description of any assumptions or corrections, such as tests of normality and adjustment for multiple comparisons
☐	☒	A full description of the statistical parameters including central tendency (e.g. means) or other basic estimates (e.g. regression coefficient) AND variation (e.g. standard deviation) or associated estimates of uncertainty (e.g. confidence intervals)
☐	☒	For null hypothesis testing, the test statistic (e.g. F, t, r) with confidence intervals, effect sizes, degrees of freedom and P value noted<br>*Give P values as exact values whenever suitable.*
☒	☐	For Bayesian analysis, information on the choice of priors and Markov chain Monte Carlo settings
☒	☐	For hierarchical and complex designs, identification of the appropriate level for tests and full reporting of outcomes
☐	☒	Estimates of effect sizes (e.g. Cohen's d, Pearson's r), indicating how they were calculated

Our web collection on statistics for biologists contains articles on many of the points above.

Software and code

Policy information about availability of computer code

Data collection	FACS-sorting: BD FACSAria III; Sequencing: Illumina NextSeq 500/550 (Illumina). Real-Time PCR: StepOne plus Real-Time PCR system (Applied Biosystem). IHC quantification: Aperio ScanScope (Leica Biosystem). Western blot acquisition: Fusion solos. Beta-galactosidase images: microscope Olympus CKX53 and nuclear quantification: ImageJ 1.52p; in vitro cell analysis: Incucyte image system and epoch for Cristal Violet quantification.
Data analysis	BD FaCSDiva v9.0, Microsoft Excel 2018. GraphPad Prism v9.0.2. ImageScope v12.3.2.8013 (Leica Biosystem). RStudio v1.3.1093. FastQC v0.11.8. Cellranger v.3.1.0 or v.6.0.0. Incucyte software v.2020B, Olympus cellSense Standard 2.3. STAR v.2.5.1b. RMagic v2.0.3. Seurat v4.1.0. clusterProfiler v4.2.2. progeny v1.16.0. GSVA v 1.42.0. stats v4.1.0. scmap v1.16.0. SingleR v1.8.1. ggplot2 v3.3.5. ggpubr v0.4.0.<br>The senescence index tool custom code is attached as supplementary data 5. The other codes are publically available and have been described in the methods section.

For manuscripts utilizing custom algorithms or software that are central to the research but not yet described in published literature, software must be made available to editors and reviewers. We strongly encourage code deposition in a community repository (e.g. GitHub). See the Nature Portfolio guidelines for submitting code & software for further information.

Data

Policy information about availability of data

All manuscripts must include a data availability statement. This statement should provide the following information, where applicable:

- Accession codes, unique identifiers, or web links for publicly available datasets
- A description of any restrictions on data availability
- For clinical datasets or third party data, please ensure that the statement adheres to our policy

In the figure legend we have added a statement regarding data availability. Source Data for the main and Extended Data Figures are provided in the manuscript. The single-cell RNA sequencing data generated in this study have been deposited in the Gene Expression Omnibus (GEO) database under accession code GSE189519 [https://www.ncbi.nlm.nih.gov/geo/query/acc.cgi?acc=GSE189519] for xenograft prostate cancer model and GSE189307 [https://www.ncbi.nlm.nih.gov/geo/query/acc.cgi?acc=GSE189307] for murine prostate cancer models. The publicly available RNA-seq data used in this study are available in GEO (Gene Expression Omnibus), and EMBL-EBI databases under accession codes GSE115301 [https://www.ncbi.nlm.nih.gov/geo/query/acc.cgi?acc=GSE115301], GSE61130 [https://www.ncbi.nlm.nih.gov/geo/query/acc.cgi?acc=GSE61130], GSE130727 [https://www.ncbi.nlm.nih.gov/geo/query/acc.cgi?acc=GSE130727], GSE102639 [https://www.ncbi.nlm.nih.gov/geo/query/acc.cgi?acc=GSE102639], GSE132369 [https://www.ncbi.nlm.nih.gov/geo/query/acc.cgi?acc=GSE132369], GSE98440 [https://www.ncbi.nlm.nih.gov/geo/query/acc.cgi?acc=GSE98440] , GSE109270 [https://www.ncbi.nlm.nih.gov/geo/query/acc.cgi?acc=GSE109270] , GSE158743 [https://www.ncbi.nlm.nih.gov/geo/query/acc.cgi?acc=GSE158743] and E-MTAB-9970 [https://www.ebi.ac.uk/arrayexpress/experiments/E-MTAB-9970]. For alignment of sc-RNA sequencing data we used mouse genome (mm10 v3.0.0) or human genome (GRCh38). All the software used for the analysis is described and referenced in the respective Method section.

Field-specific reporting

Please select the one below that is the best fit for your research. If you are not sure, read the appropriate sections before making your selection.

☒ Life sciences ☐ Behavioural & social sciences ☐ Ecological, evolutionary & environmental sciences

For a reference copy of the document with all sections, see nature.com/documents/nr-reporting-summary-flat.pdf

Life sciences study design

All studies must disclose on these points even when the disclosure is negative.

Sample size	Sample size both for in vitro and in vivo was chosen taking in consideration the means of the target values between the experimental group and the control group, the standard error and the statistical analysis used. For animal studies, sample size was defined on the basis of past experience with the models, to detect differences of 20% or greater between the groups (10% significance level and 80% power). For ethical reasons, the minimum number of animals necessary to achieve the scientific objectives was used.
Data exclusions	Grubb's test was applied to exclude outliers. We excluded some experimental replicates in Cristal Violet, RT-qPCR and SA-bGal assay.
Replication	Experiments were replicated several times with reproducible results as indicated in figure legend/Statistics and reproducibility.
Randomization	Cell lines and animals were allocated randomly to each treatment group. Different treatment groups were processed identically, and animals in different treatment groups were exposed to the same environment.
Blinding	In IHC analysis, Cristal Violet and SA-b Gal assays, the investigators were unaware of the experimental groups. The other experiments are blinindg was not possible since the same operator was responsible of the samples preparation.

Reporting for specific materials, systems and methods

We require information from authors about some types of materials, experimental systems and methods used in many studies. Here, indicate whether each material, system or method listed is relevant to your study. If you are not sure if a list item applies to your research, read the appropriate section before selecting a response.

Materials & experimental systems

n/a	Involved in the study	
☐	☒	Antibodies
☐	☒	Eukaryotic cell lines
☒	☐	Palaeontology and archaeology
☐	☒	Animals and other organisms
☒	☐	Human research participants
☒	☐	Clinical data
☒	☐	Dual use research of concern

Methods

n/a	Involved in the study	
☒	☐	ChIP-seq
☐	☒	Flow cytometry
☒	☐	MRI-based neuroimaging

Antibodies

Antibodies used

For IHC anti-Cleaved Caspase 3 (Cell signalling #9661), anti-Ki67 (RTU-Lab Vision #RM-9106-R7 Dilution Ready to use), anti-Luciferase (Abcam #ab181640), Mcl-1 (Cell signaling #5453) Ly-6G (GR1), Clone 1A8 (RUO); 551459 BD Pharmigen, F4/80 (BM8) Rat Mono, 14-4801-82 eBioscience™ (Thermo Scientific) p16 (Abcam #ab211542) were used. For Western blot anti-Cleaved Caspase 3 (Cell signalling #9664), anti-Bcl-2 (Cell signalling #3498S), anti-Mcl-1 (Cell signalling #5453) anti-HSP90 (Cell signalling #4874), p27 kip1 (Cell signalling #3698S), p15 ink4b (Abcam #53034), p16ink4a (Abcam #211542), p21 (Abcam #107099), p19 ARF (5-C3-1) (Santa Cruz Biotechnology #SC-32748), PAI-1 (Abcam #66705), Mcl-1 (Cell signaling #5453), secondary Anti-Rabbit (Promega #W4011), secondary Anti-Rat (Thermo Fisher #31470). For FACS sorting CD326 (EpCAM) Monoclonal Antibody (G8.8), FITC, eBioscience™ (11-5791-82).

Validation

All the antibodies were used accordingly with the manufacturer's instruction. More details are listed in sheet "Antibodies" present in Source Data file.

Eukaryotic cell lines

Policy information about cell lines

Cell line source(s)

HEK-293T CRL-3216™ATCC® , TRAMP-C1 cell line, ATCC® CRL-2730™; PC-3 ATCC® CRL-1435™; LNCaP clone FGC ATCC® CRL-1740™. RapidCap cell lines were obtained from Lloyd Trotman laboratory. Where indicated, cells were enginereed as described in the method sections.

Authentication

Most cell lines used were purchased from ATCC. No further authentication were performed.

Mycoplasma contamination

Cells were routinely tested for mycoplasma with MycoAlert Mycoplasma Detection kit (Lonza, Cat. LT07-218) and only mycoplasma free cells were used.

Commonly misidentified lines
(See ICLAC register)

No commonly misidentified cell lines were used in the study.

Animals and other organisms

Policy information about studies involving animals; ARRIVE guidelines recommended for reporting animal research

Laboratory animals

All mice were maintained under specific pathogen-free conditions in the IRB facility (Bellinzona, Switzerland). Experiments were performed according to the state guidelines and approved by the local ethical committee (‘‘Dipartimento della Sanità e Socialità, Esperimenti su animali’’ Canton Ticino), authorization number TI-51/2018 (Maximum tumor volume authorized = 1500 mm3, not exceeded). Male NOD/SCID mice were purchased from Charles River (Calco, Italy) and acclimatized for four weeks before experimentation (xenograft models), as described in methods sections.
Ptenpc-/- mice were generated and genotyped as follow: female Pten loxP/loxP mice were crossed with male Pten loxP/WT;PB-Cre4 transgenic mice and genotyped for Cre using following primers: primer 1 (5'-AAAAGTTCCCCTGCTGATGATTTGT-3') and primer 2 (5'-TGTTTTTGACCAATTAAAGTAGGCTGTG-3') and for PTENloxP/loxP: primer1 (5’ TGATGGACATGTTCAGGGATC 3’) and primer2 (5’CAGCCACCAGCTTGCATGA 3’) for Probasin-CRE. Prostate-specific Ptenpc-/- transgenic mice were crossed with Timp1-/- mice (Jackson Laboratory, 6243) to generate Timp1 knock out in Ptenpc-/-.
Mice undergoing treatment were administered control vehicle or therapeutic doses of the appropriate agents. Any mouse suffering distress or greater than 15% weight loss during treatment was euthanized by CO2 asphyxiation. At the completion of study, mice were euthanized by CO2 asphyxiation and tissue were collected for histology (paraffin embedded and OCT embedded), mRNA analysis, western blots and single cell suspensions for 10x Genomics sequencing.
Mice used for 10x scRNA sequencing were euthanized at 10 weeks of age. NRG mice have been challenged with PC3 shTimp1 at 8 weeks of age and then monitored and kept under treatment up to 42 days post-injection. Finally Ptenpc-/- mice have been treated at 10 weeks of age and euthanized upon 6 weeks of treatment.

Wild animals

No wild animals were used in this study.

Field-collected samples

No field collected samples were used in the study.

Ethics oversight

The animal experiments were approved by the local ethical committee by the local ethical committee (‘‘Dipartimento della Sanità e Socialità, Esperimenti su animali’’ Canton Ticino), authorization number TI-51/2018 .

Note that full information on the approval of the study protocol must also be provided in the manuscript.

Flow Cytometry

Plots

Confirm that:

☒ The axis labels state the marker and fluorochrome used (e.g. CD4-FITC).

☒ The axis scales are clearly visible. Include numbers along axes only for bottom left plot of group (a 'group' is an analysis of identical markers).

☒ All plots are contour plots with outliers or pseudocolor plots.

☐ A numerical value for number of cells or percentage (with statistics) is provided.

Methodology

Sample preparation	Prostate tumors or xenografts tumors were isolated, minced and processed for single cell suspension. Tissues were digested in 2 ml of Digestion Buffer composed by RPMI 10% FBS + 1% P/S, 500 mL of Collagenase D (1 mg/mL), 50mL of DNAse (100 U/mL) and 125mL of HEPES (25 mM). The cell suspension was incubated for 50 minutes at 37°C on a rocker. Then, the digestion was stopped adding 1 mL of RPMI 10% FBS + 1% P/S. The cells suspension was filtered through a 100 um cell strainer and kept on ice for 4 minutes. Then cells suspension was filtered again through a 40 °m cell strainer and spun down at 453g for 5 min at 4°C. FACS staining was performed using EPCAM-FITC (anti-Mo CD326, eBioscience, clone G8.8 #11-5791-82) for the murine prostate tumors, while PC3 shTIMP1 were sorted thanks to GFP positivity.
Instrument	Samples were acquired on a BD sorter Aria III (BD Biosciences).
Software	The software used was BD FaCSDiva v9.0. No further analysis were needed for this study.
Cell population abundance	The abundancy of EPCAM positive cells soterd in Suppl. Fig1 was in a range of 54 to 63%. While the sorting of the GFP positive cells shown in Suppl Fig7 was from 35 to 55%.
Gating strategy	The gating strategy used in Suppl. Fig1 have been done as it follows: FSC-H/FSC-A, SSC-A/FSC-A, 7AAD/FSC-A, EPCAM+/FSC-A. While Suppl. Fig7 FSC-H/FSC-A, SSC-A/FSC-A, GFP+/FSC-A. Further information are displayed in the figures.

☒ Tick this box to confirm that a figure exemplifying the gating strategy is provided in the Supplementary Information.
