# Supplementary material for: Single-cell transcriptomics identifies Mcl-1 as a target for senolytic therapy in cancer
Source: Nat Commun. 2022 Apr 21;13:2177. doi: 10.1038/s41467-022-29824-1 (PMC9023465; doi:10.1038/s41467-022-29824-1)
Supplement: Supplementary file 1 — Supplementary Information [file 41467_2022_29824_MOESM1_ESM.pdf]

Supplementary Information

Single-cell transcriptomics identifies Mcl-1 as a target for senolytic therapy in cancer

Martina Troiani[1,2,3,12], Manuel Colucci[1,2,4,12], Mariantonietta D'Ambrosio[1,2,4], Ilaria Guccini[5], Emiliano Pasquini[1,2], Angelica Varesi[1,2], Aurora Valdata[1,2], Simone Mosole[1,2], Ajinkya Revandkar[1,2,6], Giuseppe Attanasio[1,2], Andrea Rinaldi[1,2], Anna Rinaldi[7,8], Marco Bolis[1,2,3,9], Pietro Cippà[7,8] and Andrea Alimonti[1,2,10,11] *.

[1]Institute of Oncology Research (IOR), Oncology Institute of Southern Switzerland (IOSI), Bellinzona CH6500, Switzerland.

[2]Universita' della Svizzera Italiana, Lugano CH6900, Switzerland

[3]Bioinformatics Core Unit, Swiss Institute of Bioinformatics, TI 6500 Bellinzona, Switzerland

[4]Faculty of Biology and Medicine, University of Lausanne UNIL, Lausanne CH1011, Switzerland

[5]Institute of Molecular Health Sciences, ETH Zurich, Zurich 8093, Switzerland

[6]Massachusetts General Hospital Cancer Center, Harvard Medical School, Charlestown, MA 02129, USA.

[7]Department of Medicine, Division of Nephrology, Ente Ospedaliero Cantonale, Lugano, Switzerland

[8]Laboratories for Translational Research, Ente Ospedaliero Cantonale, Bellinzona, Switzerland

[9]Computational Oncology Unit, Department of Oncology, Istituto di Ricerche Farmacologiche 'Mario Negri' IRCCS, 20156 Milano, Italy

[10]Department of Health Sciences and Technology (D-HEST) ETH Zurich, Zurich CH 8093, Switzerland.

[11]University of Padova, Department of Medicine & Veneto Institute of Molecular Medicine, Padova, Italy

[12]These authors contributed equally

Contents:

Supplementary Figures 1-9

Supplementary Table 1-2

Supplementary Figure 1

a

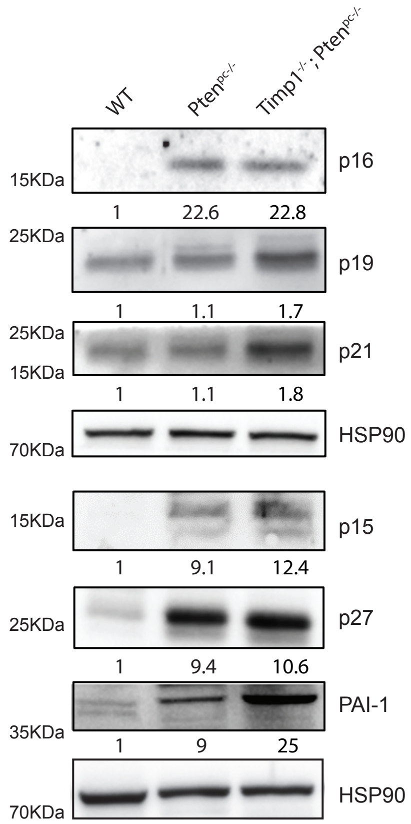

b

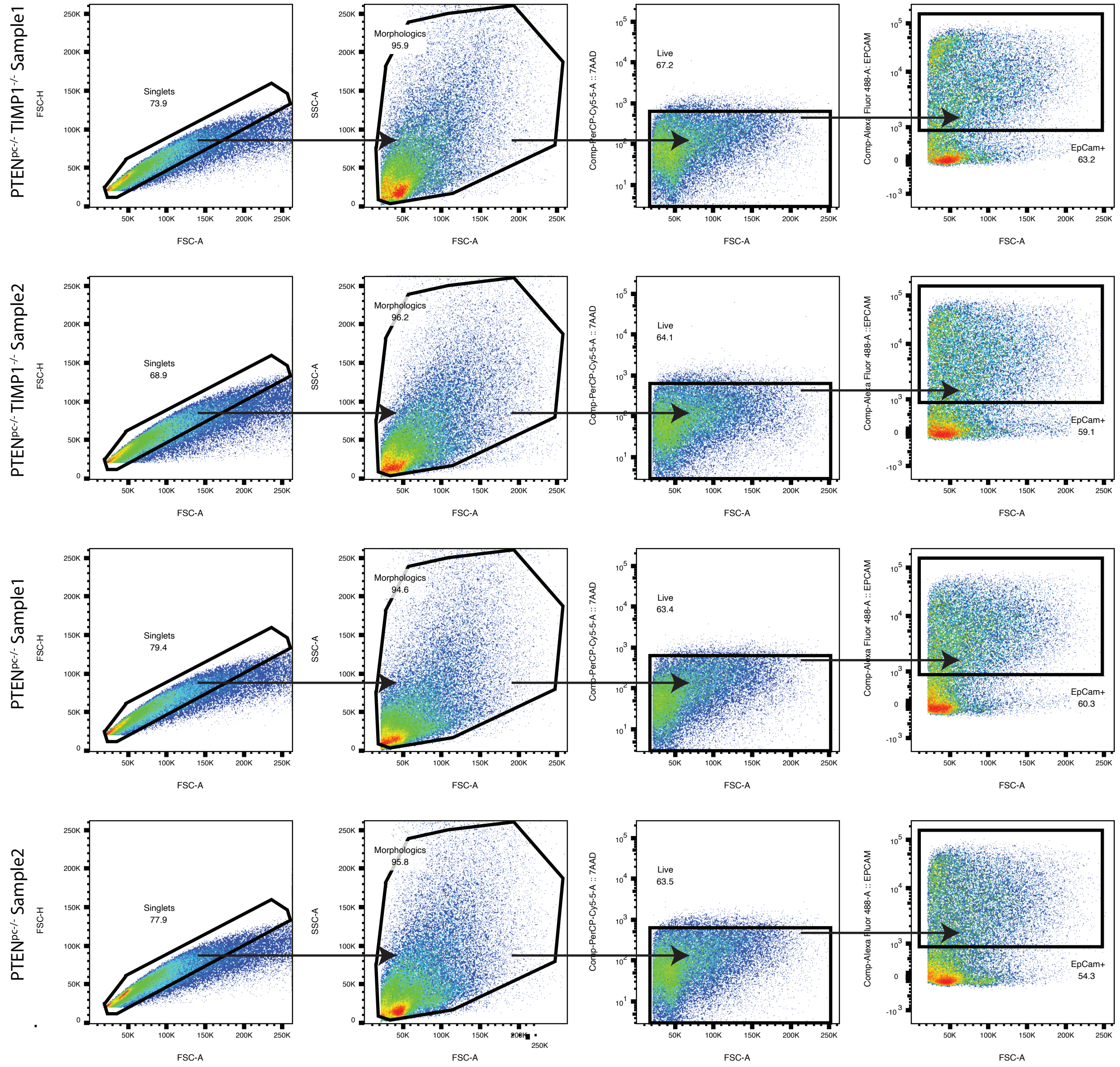

Supplementary Figure1. Markers of senescence in prostate cancer and sorting strategy for 10X Genomics analysis. a, Western blots with quantification for the senescence markers (*$p16^{INK4a}$, $p15^{INK4b}$, $p19^{Arf}$, $P21^{Waf1/Cip1}$, $p27^{Kip1}$* and *PAI-1*) in the indicated genotypes. Data are representative of three independent experiments. **b**, Gating strategy of the epithelial tumor cells of the different genotypes identified by the EPCaM antibody. Data are representative of 4 animals from one experiment.

Supplementary Figure 2

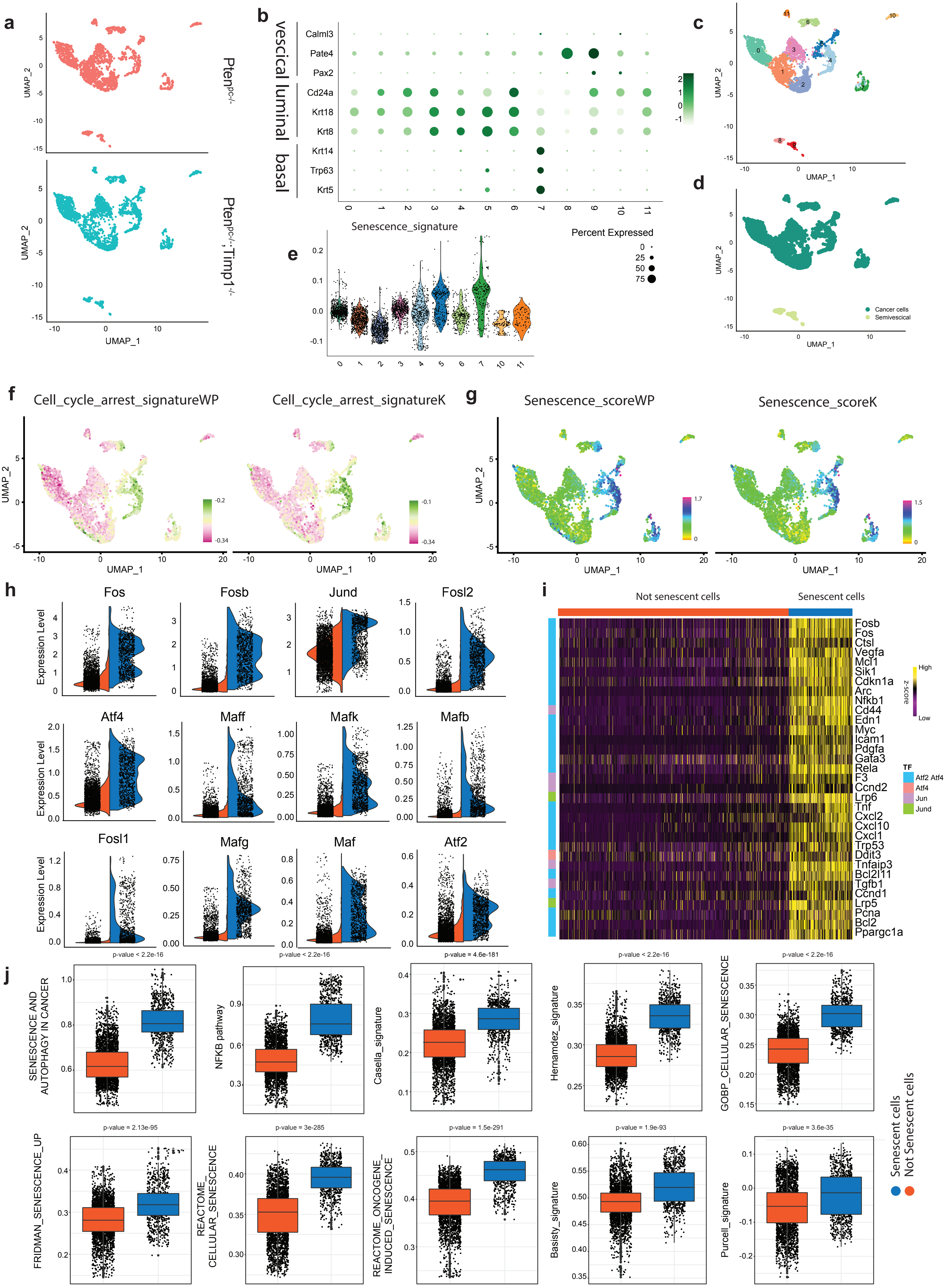

Supplementary Figure2. Senescence definition at single cells level. a, UMAP plots of 10X Genomics integrated data of *Pten*$^{pc-/-}$ and *Pten*$^{pc-/-}$; *Timp1*$^{-/-}$ mouse models. **b**, Epithelial marker genes expressions for each cluster of the 12 ones found with *FindClusters* function. Semi-vescical markers: Calml3, Pate4, Pax2. Luminal markers: Krt18, Krt8, Cd24a. Basal markers: Krt14, Trp63, Krt5. **c**, UMAP plot showing 12 clusters found with *FindClusters* function. **d**, UMAP plot of classification of cells as cancer cells and semi-vescical cells. **e**, Violin plot of Senescent signature between the 10 cancer cell clusters. **f**, UMAP plot of cancer epithelial cells colored by Cell_cycle_arrest_signatureWP and signatureK. **g**, UMAP plot of cancer epithelial cells colored by Senescence_scoreWP (SWP) and Senescence_scoreK (SK). **h**, Violin plot of the up-regulated genes in senescent cells belonging to the AP-1 TF family. **i**, Heatmap of target genes of TF upregulated in senescent cells. **j**, Boxplot of ss-GSVA scores for gene sets related to senescence: Senescence and autophagy in cancer (Wikipathway), NFKB pathway (Biocarta), Casella_signature, Hernandez_signature, Fridman_senescence_up, Basisty_signature, Purcell_signature, Cellular senescence from Gene Ontology collection, Oncogene-induced senescence and Cellular senescence genesets from Reactome collection. (N= 3992 cells, two-sided Wilcox-test: Senescence and autophagy in cancer p-value < 2.2e-16; Not senescent cells (left) median = 0.617 Senescent cells (right) median = 0.806; NFKB pathway p-value < 2.2e-16; Not senescent cells (left) median = 0.472, Senescent cells (right median = 0.757; Casella_signature p-value = 4.6e-181; Not senescent cells (left) median = 0.226, Senescent cells (right) median = 0.287; Hernandez_signature p-value < 2.2e-16; Not senescent cells (left) median = 0.286, Senescent cells (right) median = 0.335; GO_cellular_senescence p-value < 2.2e-16; Not senescent cells (left) median = 0.242, Senescent cells (right) median = 0.302; Fridman_senescence_up p-value = 2.13e-95; Not senescent cells (left) median = 0.28, Senescent cells (right) median = 0.318; Reactome_cellular_senescence p-value = 3e-285; Not senescent cells (left) median = 0.353, Senescent cells (right) median = 0.396; Reactome oncogene_induced_senescence p-value = 1.5e-291; Not senescent cells (left) median = 0.396, Senescent cells (right) median = 0.462; Basisty_signature p-value = 1.9e-93; Not senescent cells (left) median = 0.493, Senescent cells (right) median = 0.52; Purcell_signature p-value = 3.6e-35; Not senescent cells (left) median = -0.053, Senescent cells (right) median = -0.013. Source data are provided as a Source Data file.

Supplementary Figure 3

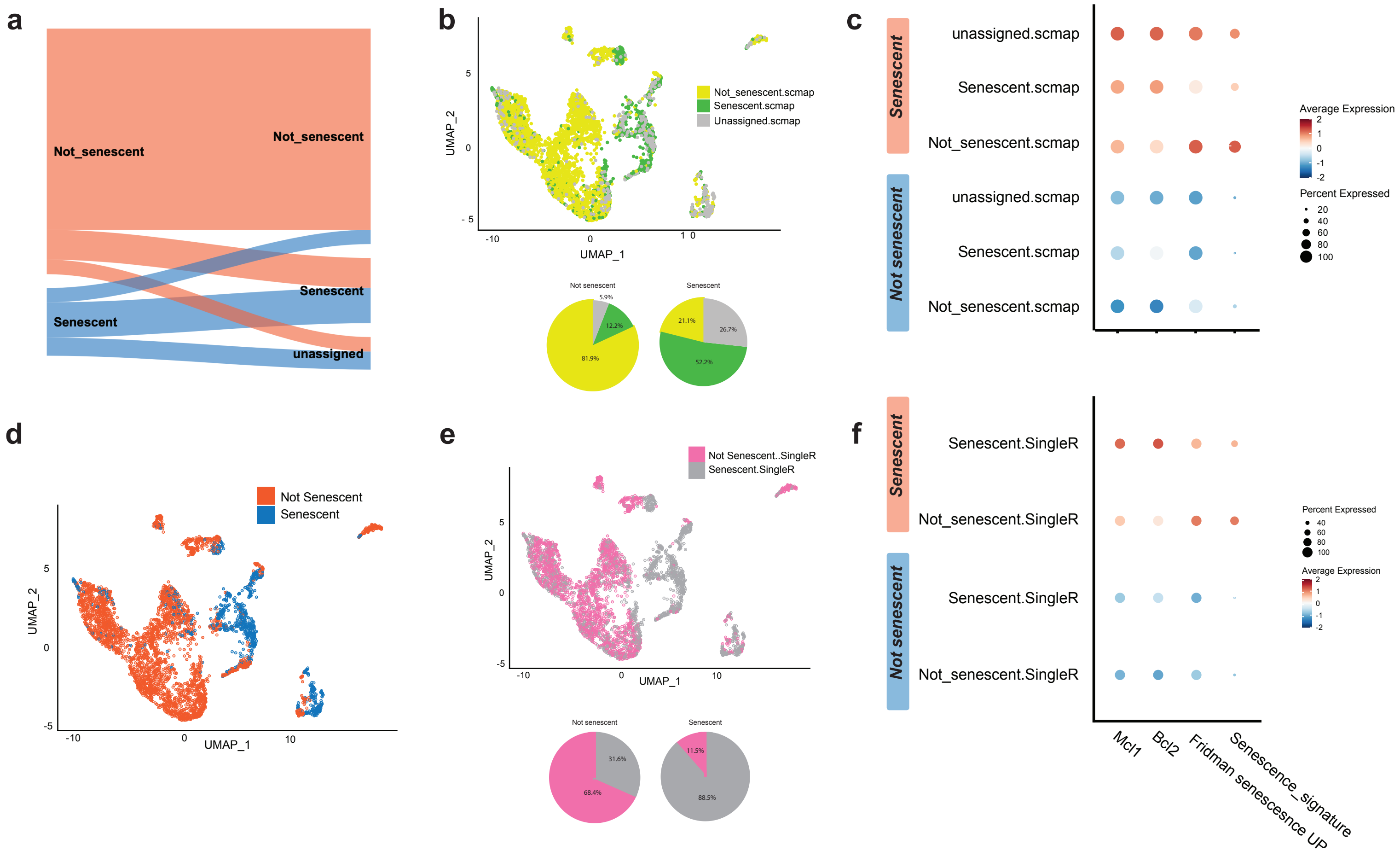

Supplementary Figure3. Out-of-clustering analysis for validation of Senescence Index Tool. a, Scmap cluster projection of the SIT classification to the senescence datasets of Supplementary Table 1. **b,** UMAP plot showing the scmap results. Yellow dots = not senescent cell for scmap (N= 2746 cells); green dots = senescent cells for scmap (N= 831 cells); grey dots = unassigned cells for scmap (N=415 cells). (Bottom) Pie-charts of percentage of scmap results in SIT classification: 52% of senescent tumor cells recognized by the SIT are also recognized as senescent by the scmap. 26% of cells classified as senescent using the SIT, results unassigned with the scmap, but upregulated canonical senescent markers, the Fridman_senescence signature, Mcl1 and Bcl2. **c,** Dotplot showing expression of Mcl1, Bcl2, Fridman_senescence signature and Senescence signature in scmap result. **d,** UMAP plot of cancer epithelial cells showing senescent cells (blue) and not senescent cells (orange). **e,** UMAP plot showing the SingleR results. Pink dots = Not senescent cells for SingleR (N= 2243 cells); grey dots = Senescent cells for SingleR (N=1749 cells). (Bottom) Pie-charts of percentage of SingleR results in SIT classification: 88.5% of senescent tumor cells recognized by the SIT is also categorized as senescence by using SingleR. **f,** Dotplot showing expression of Mcl1, Bcl2, Fridman_senescence signature and Senescence signature in SingleR results.

Supplementary Figure 4

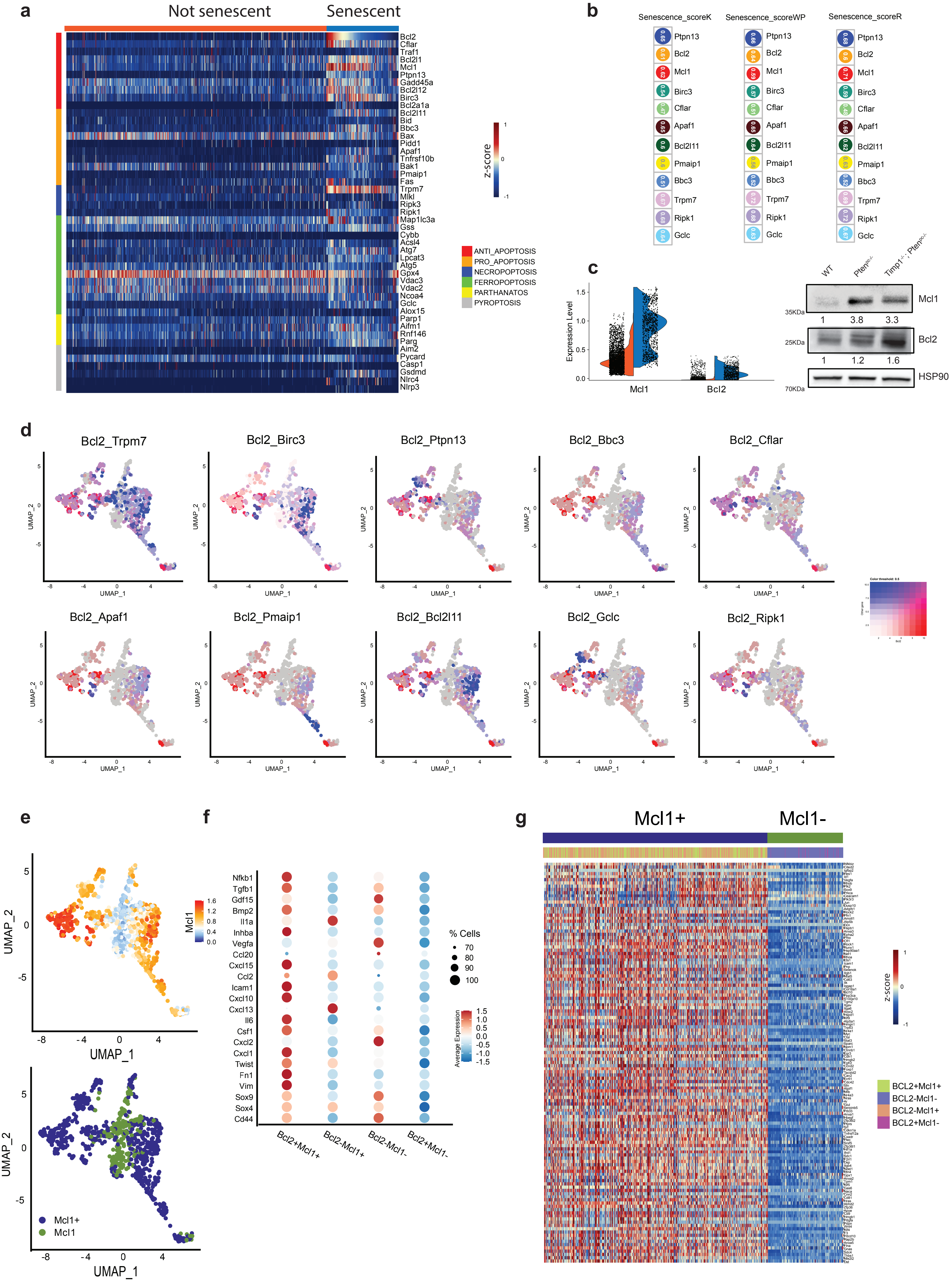

Supplementary Figure4. Cell-death related genes and senescence. a, Heatmap of 47 cell death related-genes between senescent and not senescent cells. **b**, Correlation matrix showing Pearson correlation coefficients (R higher than 0.4) between cell death-related genes and Senescence_scoreK, Senescence_scoreWP and Senescence_scoreR. Only genes with FDR<0.05 are reported. **c**, Left panel, violin plot of Mcl-1 and Bcl2 in senescent cells and not senescent cells. Right panel, Western blots for Mcl-1 and Bcl2 in the three different genotypes. Western blot data are representative of three experiments. **d**, Co-expression of Bcl2 and selected cell death-related genes in senescent cells. Red cells mostly express Bcl2, blue cells mostly express the gene of interest while purple cells co-express both targets. **e**, UMAP plot showing Mcl1 expression levels in senescent cells (top) and classification of them in Mcl-1^+ or Mcl-1^- (bottom). **f**, Dotplot showing SASP genes and metastatic related genes between the different senescent subpopulations. **g**, Heatmap of differentially expressed genes related to migration, mobility and locomotion in senescent cells Mcl-1^+ and Mcl-1^-. Source data are provided as a Source Data file.

Supplementary Figure 5

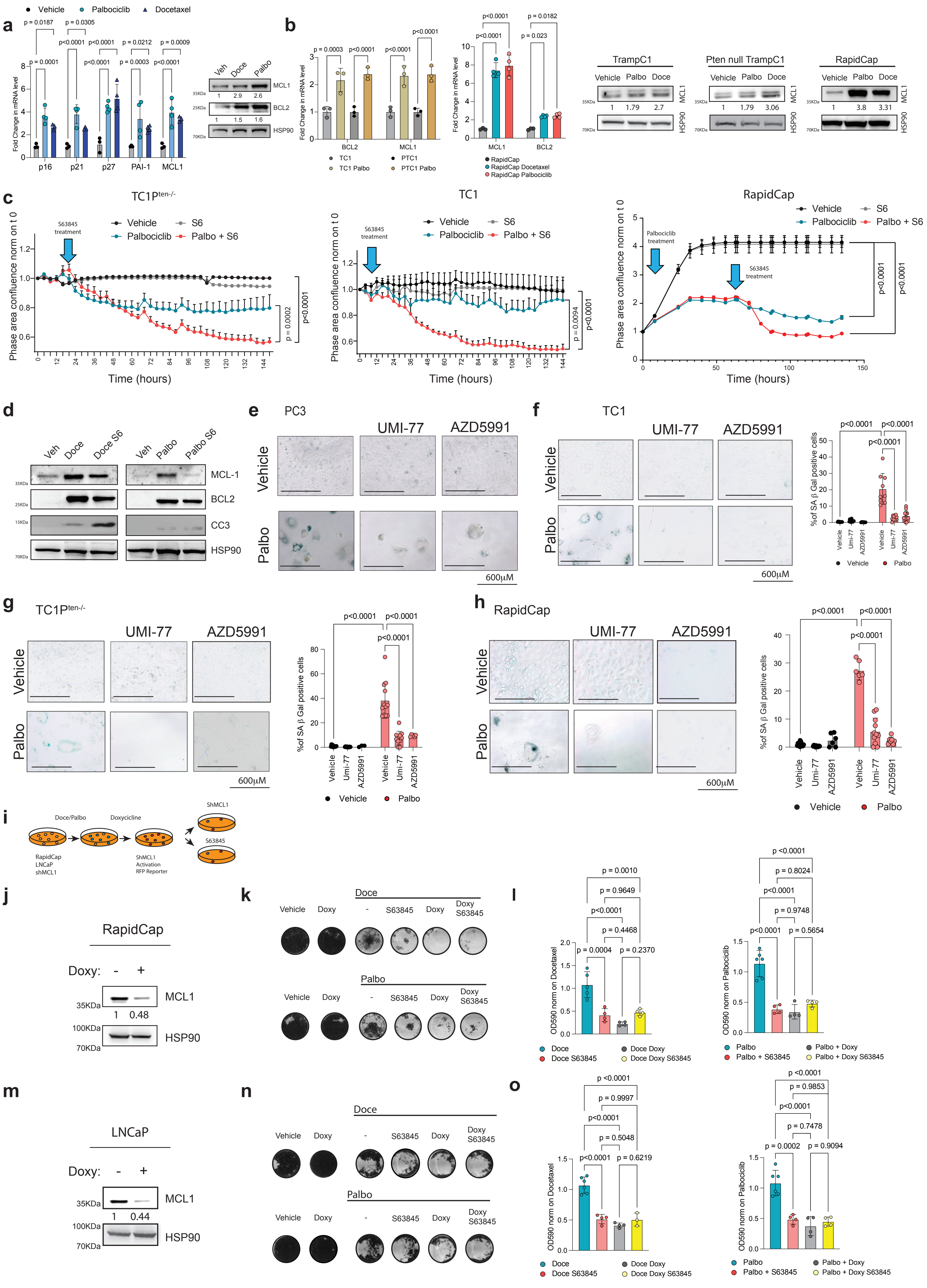

Supplementary Figure5. Validation of senolytic activity of MCL-1 inhibition with pharmacological and genetic approaches. a, RT-qPCR for senescence markers and western blot of pro-survival proteins of PC3 shTIMP1 upon TIS (for Vehicle sample n=3 for each gene, for Palbociclib n = 4 for each gene, for Docetaxel n = 3 for p16, p21 and MCL1 while n = 4 for p27 and PAI-1, n= biological independent replicates from two experiments). **b,** RT-qPCR and western blots of senescent murine cell lines (TC1, $TC1^{Pten-/-}$ and RapidCap) for Mcl-1 (n=3 for Vehicle, Docetaxel and Palbociclib in TC1, $TC1^{Pten-/-}$ and n=3 for Vehicle and n =4 for Docetaxel and Palbociclib in RapidCap, n= biological independent replicates from two experiments) and for Bcl2 (n=3 for Vehicle, Docetaxel and Palbociclib in TC1, $TC1^{Pten-/-}$ and n=3 for Vehicle and n =4 for Docetaxel and Palbociclib in RapidCap, n= biological independent replicates from two experiments). **c,** Incucyte Growth curve of TC1, $TC1^{Pten-/-}$ and RapidCap subjected to Palbociclib first and S63845 after (in TC1 n=7 for Vehicle, n= 3 for Palbociclib and S63845 and n = 5 for Palbociclib + S63845; in $TC1^{Pten-/-}$ n=6 for Vehicle and Palbociclib and n = 3 for Palbociclib + S63845 and S63845). **d,** Western blots of PC3 shTIMP1 treated with TIS and S63845. Data are representative of one experiment. **e,** Representative images of SA-β–Gal of PC3. Data are representative of two experiments. **f,** Representative images of SA-β–Gal of TC1 treated with TIS and two different MCL1 inhibitors and its quantification (in Vehicle category (black dots) n=12 for Vehicle and UMI-77 , n = 8 for AZD5991; in Palbociclib treatment (pink dots) n=9 for Palbociclib, n= 11 for UMI-77 and n=12 for AZD5991 ; n=biological independent replicates from three experiments); **g,** Representative images of SA-β–Gal of $TC1^{Pten-/-}$ treated with TIS and two different MCL1 inhibitors and its quantification (in Vehicle category (black dots) n=11 for Vehicle, n = 12 for UMI-77, n = 3 for AZD5991; in Palbociclib treatment (pink dots) n=12 for Palbociclib, n= 11 for UMI-77 and n=6 for AZD5991) ; n=biological independent replicates from three experiments). **h,** Representative images of SA-β–Gal of RapidCap treated with TIS and two different MCL1 inhibitors and its quantification (in Vehicle category (black dots) n=12 for Vehicle and UMI-77, n = 6 for AZD5991; in Palbociclib treatment (pink dots) n=6 for Palbociclib, n= 11 for UMI-77 and n=7 for AZD5991) ; n=biological independent replicates from three experiments); **i,** Experimental design. **j,** Western blot analysis of shMCL1 in the murine RapidCap cells, and its quantification. Data are representative of two experiments. **k,** Representative pictures of crystal violet images of the treated RapidCap cells. Data are representative of two experiments. **l,** Fold change of crystal violet quantification in RapidCap cells subjected to Docetaxel treatment (left; n=5 for Docetaxel and n=4 for Docetaxel S63845, Docetaxel Doxy and Docetaxel Doxy S63845) and to Palbociclib (right; n= 6 for Palbociclib and n=4 for Palbociclib S63845, Palbociclib Doxy and Palbociclib Doxy S63845; n= biological independent replicates from two experiments). **m,** Western blot of shMCL1 in the human LNCaP cells, and in its quantification. Data are representative of two experiments. **n,** Representative pictures of crystal violet images of the treated LNCaP cells. Data are representative of two experiments. **o,** Fold change of crystal violet quantification in LNCaP cells to Docetaxel treatment (left; n=6 for Docetaxel, n=4 for Docetaxel S63845 and Docetaxel Doxy and n= 3 for Docetaxel Doxy S63845) and to Palbociclib (right; n= 6 for Palbociclib and n=4 for Palbociclib S63845, Palbociclib Doxy and Palbociclib Doxy S63845; n= biological independent replicates from two experiments). **a,b,f,g,h,l,o,** Data are represented as mean ± SD. **c,** Data are represented as mean ± SEM. **a,b,c,f,g,h,** The p-values were determined by two Way Anova followed by Tukey's multiple comparisons test. **l,o,** The p-values were determined by one Way Anova followed by Tukey's multiple comparisons test. The whole Anova results were given in the Supplementary Data 4. Source data are provided as a Source Data file.

Supplementary Figure 6

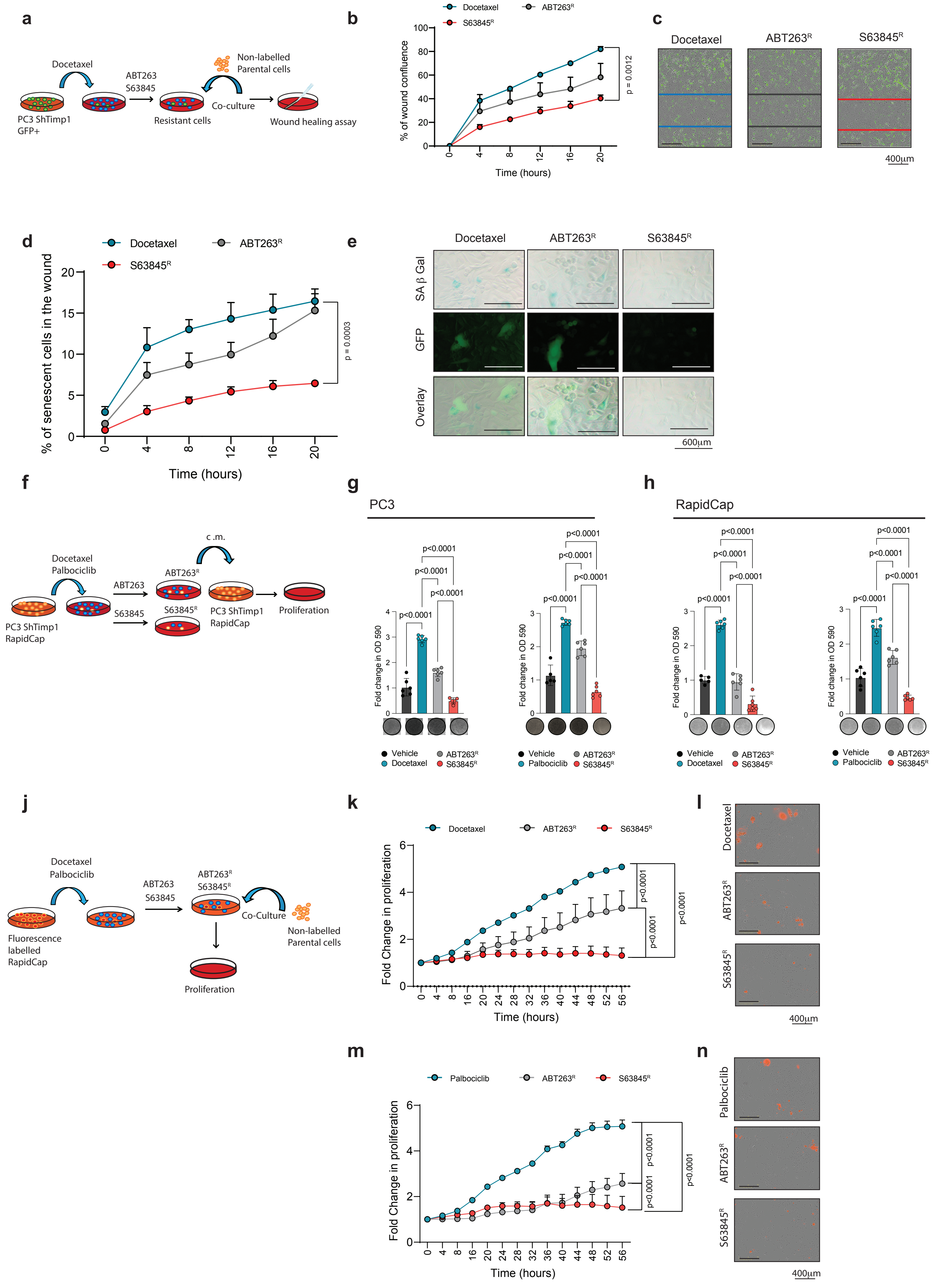

Supplementary Figure6. Mcl-1 inhibition revert proliferation and migration driven by the SASP. a Schematic representation of experimental design. **b**, Percentage of wound closure measured by Incucyte (n=2 for Docetaxel, n=4 for S63845^R and ABT26 3^R; n=biological independent replicates from two experiments). **c**, Representative pictures of wound closure upon 20 hours. Scale bar 400 µm. Data are representative of two experiments. **d**, Graph showing the percentage of senescent GFP$^+$ cells into the wound (n=2 for Docetaxel, n=3 for S63845^R and n=4 for ABT263^R; n=biological independent replicates from one experiment). **e**, Representative images showing in bright field the SA-β Gal positive cells, in fluorescence channel the positivity to GFP and the merge of the two channels. Scale bar 600µm. Data are representative of one experiment. **f**, Schematic representation of experimental design. **g**, Bar graph representing the fold change of crystal violet absorbance, and representative images below in PC3 shTIMP1 after TIS with Docetaxel (left panel n=6 for Vehicle, Docetaxel, and ABT263^R, n=5 for S63845^R; n=biological independent replicates from two experiment) and Palbociclib (right panel n=6 for Vehicle, Palbociclib, ABT263^R and S63845^R; n=biological independent replicates from two experiments) and **h**, RapidCap after TIS with Docetaxel (left panel n=5 for Vehicle, n=6 for Docetaxel, ABT263^R, and S63845^R; n=biological independent replicates from two experiments) and Palbociclib (right panel n=6 for Vehicle, Palbociclib, ABT263^R and S63845^R; n=biological independent replicates from two experiments). **j,** Iconographic representation of experiment layout. **k**, Fold change in proliferation of parental RapidCap upon co-culture of Docetaxel treated cells (n=6 for Docetaxel and S63845^R, n=5 for ABT263^R; n=biological independent replicates from two experiments). **l**, Representative images of co-culture of Docetaxel treated Tomato$^+$-RapidCap and parental untreated and un-labelled cells. Scale bar 400 µm. Data are representative of two experiments. **m**, Fold change in proliferation of parental RapidCap after co-culture with Palbociclib treated cells (n=9 for Palbocicblib, n=5 for S63845^R and n=3 for ABT263^R, n=biological independent replicates from two experiments). **n**, Representative images of co-culture with Palbociclib treated Tomato$^+$-RapidCap and parental un-labelled cells. Scale bar 400 µm. Data are representative of two experiments. Source data are provided as a Source Data file. **b,d,k,m,** Data are represented as mean ± SEM. **b,d,k,m,** The p-values were determined by two Way Anova followed by Tukey's multiple comparisons test; **g,h,** The p-values were determined by one Way Anova followed by Tukey's multiple comparisons test; the whole Anova results were given in the Supplementary Data 4.

Supplementary Figure 7

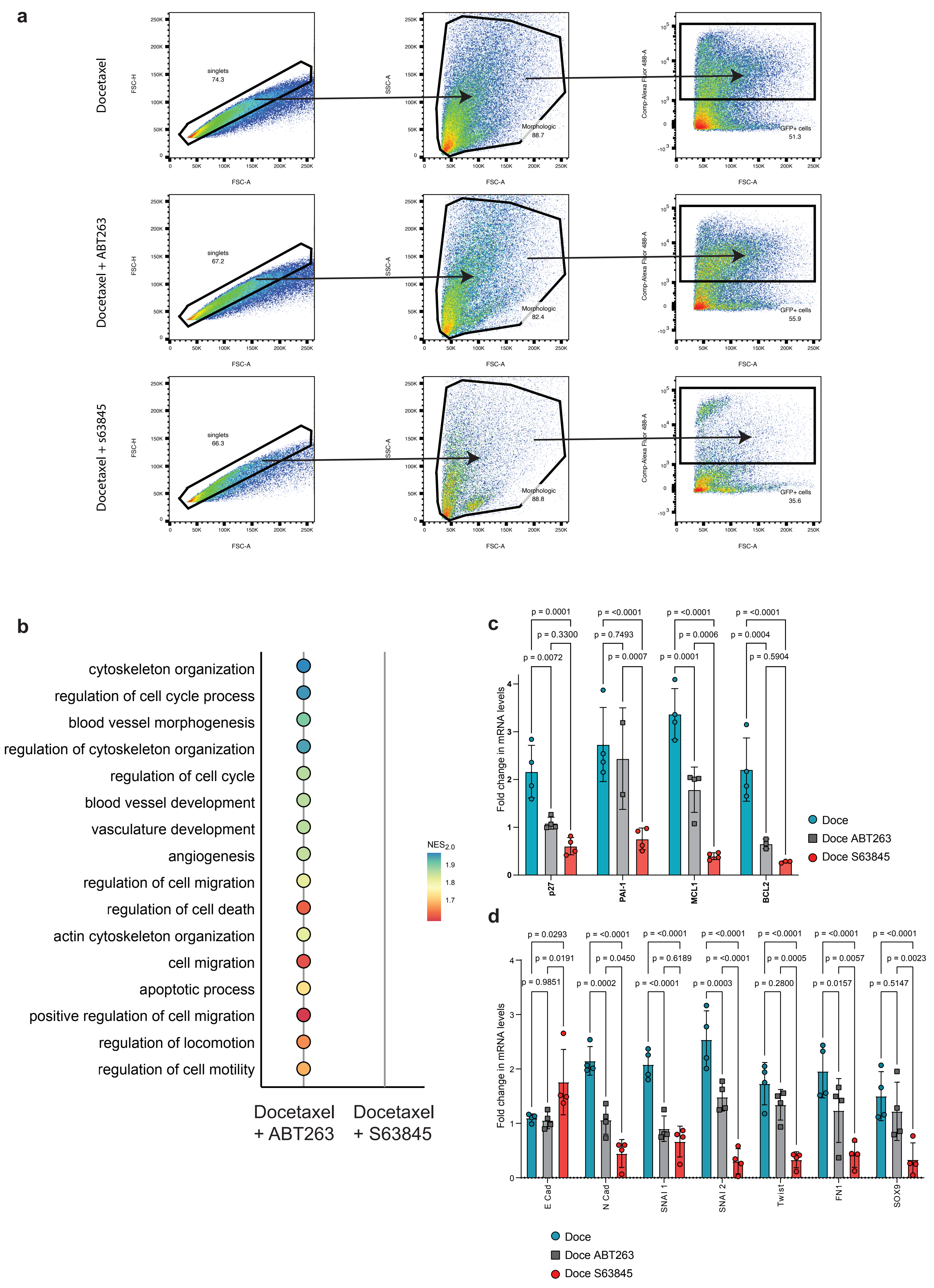

Supplementary Figure7. Removal of pro-metastatic features upon S63845 treatment. a, Gating strategy for the sorting of tumor cells isolated by using the GFP constitutively expressed by PC3shTIMP1 cell line. Data are representative of one experiment. **b,** Dotplot showing enrichment results of biological process (GO) pathway analysis by considering senescent cell treated with S63845 or ABT263. Color scale reports normalized enrichment score (NES). **c**, RT-qPCR for transcriptional markers of senescence (for p27 and MCL-1 n=4 for Docetaxel, Docetaxel + ABT263 and Docetaxel + S63845; for PAI-1 n=4 for Docetaxel and Docetaxel + S63845 and n=2 Docetaxel + ABT263; for BCL2 n=4 for Docetaxel and n =3 for Docetaxel + S63845 and Docetaxel + ABT263; n=biological independent replicates from one experiment). **d**, RT-qPCR for pro-migratory features (n=4 for Docetaxel, Docetaxel + ABT263 and Docetaxel + S63845; n=biological independent replicates from one experiment). **c,d,** The p-values were determined by Two Way Anova followed by Tukey's multiple comparisons test; the whole Anova results were given in the Supplementary Data 4. **c,d,** Data are represented as mean ± SD. Source data are provided as a Source Data file.

Supplementary Figure 8

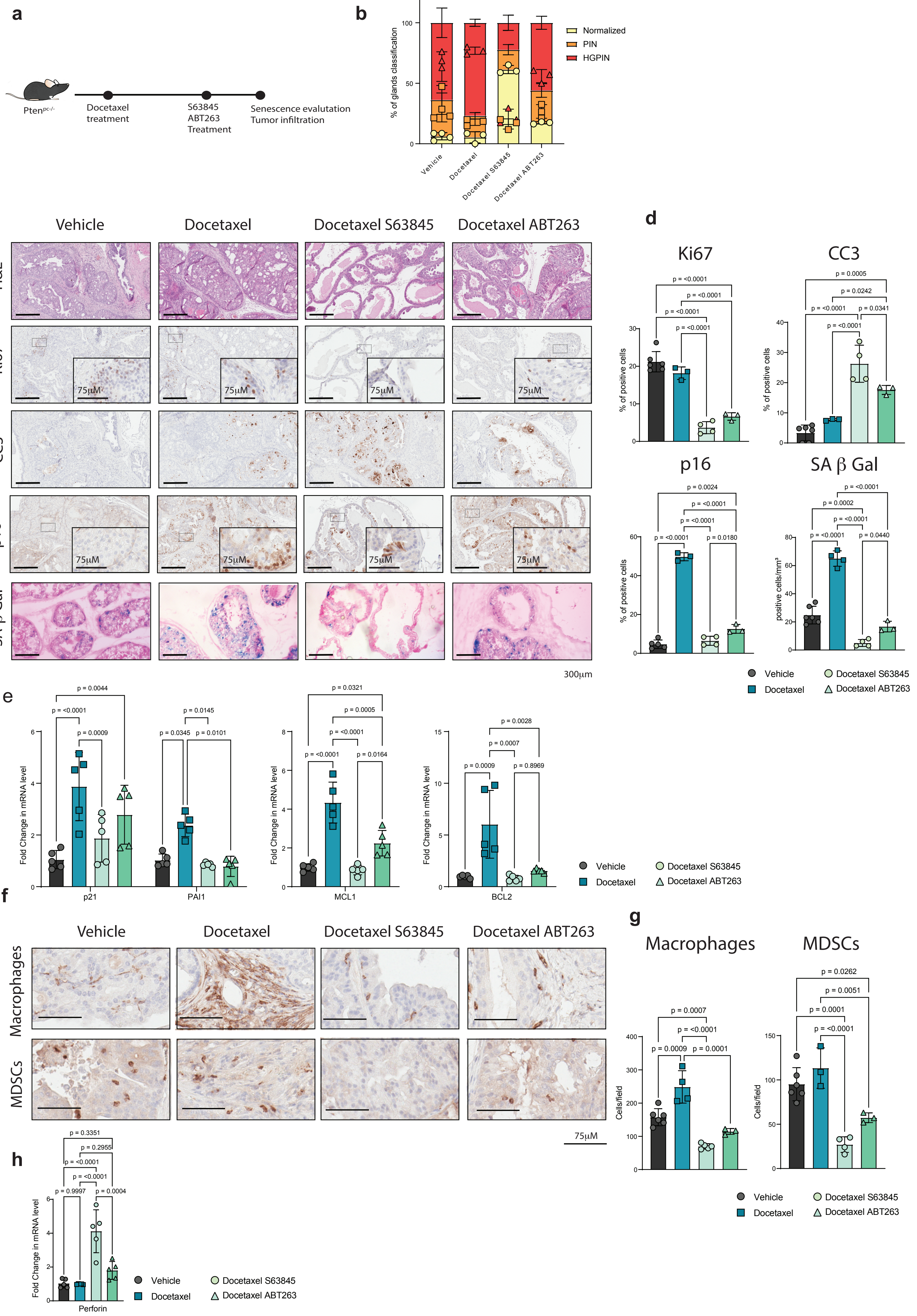

Supplementary Figure8. Senolytic treatment in transgenic *Pten$^{pc-/-}$* prostate cancer model. a, Schematic representation of the experimental design. **b,** Gland scoring expressed in percentage in the different treatment (n= 4 for Vehicle, n = 3 for Docetaxel, Docetaxel + ABT263 and Docetaxel + S63845, n= independent animals from one experiment). **c,** Representative pictures of the immunohistochemical analysis of H&E, proliferation marker Ki-67, apoptotic marker Cleaved Caspase 3 (CC3), and senescence markers p16 and SA-β Gal (Scale bar 300μm; insert 75μm). Data are representative of one experiment. **d,** Immunohistochemistry and SA-β Gal quantification (for Ki67 n= 6 for Vehicle, n=3 for Docetaxel and Docetaxel ABT263, n=4 for Docetaxel S63845, for CC3 n= 6 for Vehicle, n=3 for Docetaxel and Docetaxel ABT263, n=4 for Docetaxel S63845, for p16 n= 5 for Vehicle, n=3 for Docetaxel and Docetaxel ABT263, n=4 for Docetaxel S63845, for SA-β-Gal n= 6 for Vehicle, n=3 for Docetaxel ABT263, n=4 for Docetaxel and Docetaxel S63845, n= areas from 5 animals from one experiment). **e,** mRNA levels of marker of senescence (p21 and PAI-1) and the pro-survival factors (Mcl-1 and Bcl2) (for all genes and all conditions n=5 biological samples from 5 independent animals from one experiment). **f,** Representative pictures of immunohistochemical analysis of the immune subpopulations: Macrophages (F4/80) and Myeloid-derived suppressor cells (Gr1). Data are representative of one experiment. **g,** Quantification of immunohistochemistry of panel f (for macrophages n= 6 for Vehicle, n=3 for Docetaxel, n= 3 for Docetaxel ABT263, n= 5 for Docetaxel S63845, for Myeloid-derived suppressor cells n= 6 for Vehicle, n=3 for Docetaxel and Docetaxel ABT263, n=4 for Docetaxel S63845, n= areas from 5 animals from one experiment). **h,** mRNA levels of Perforin expression (n=5 biological replicates from 5 independent animals from one experiment). **d,e,g,h,** The p-values were determined by One Way Anova followed by Tukey’s multiple comparisons test. The whole Anova results were given in the Supplementary Data 4. **b,d,e,g,h,** Data are represented as mean ± SD. Source data are provided as a Source Data file.

Supplementary Figure 9

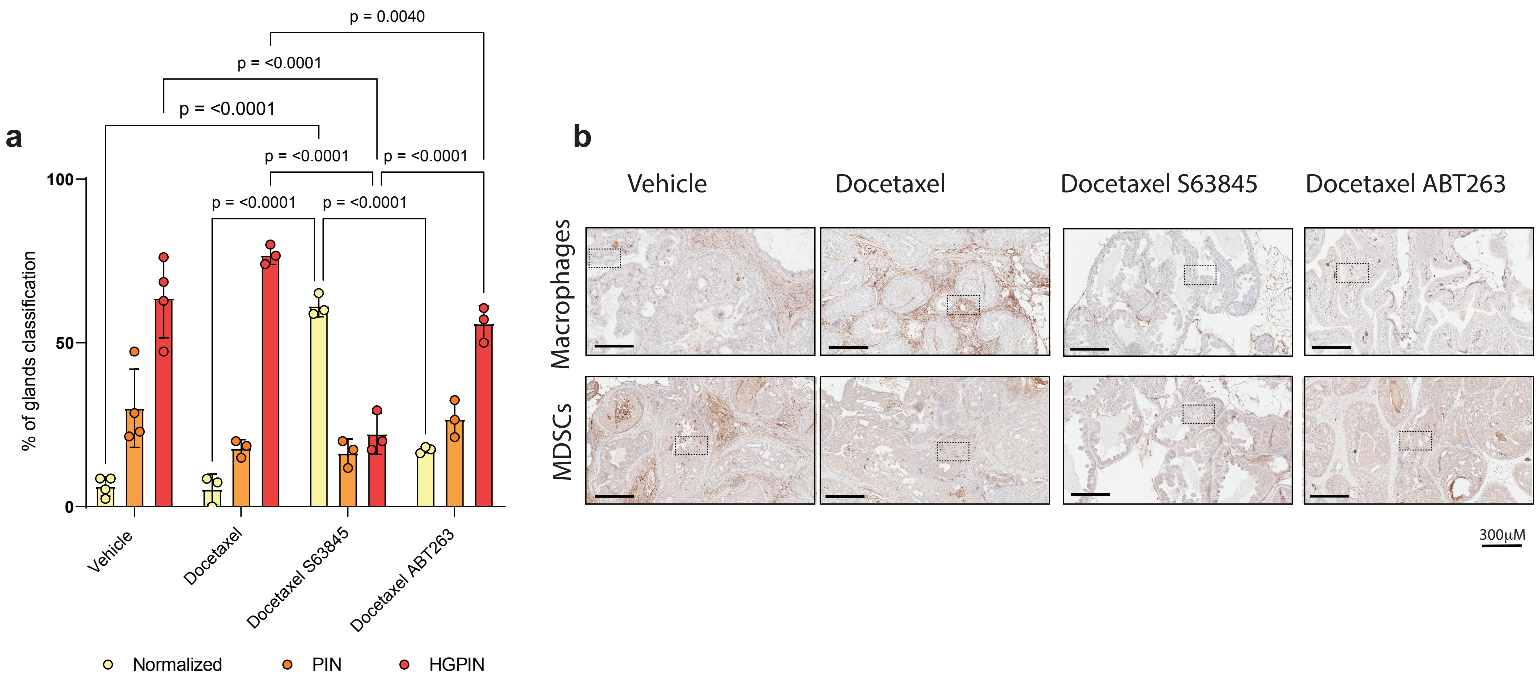

Supplementary Figure9. S63845 treatment have an effect on prostate gland and the immune infiltrate. **a,** % of glands scoring in the different treatments (n= 4 for Vehicle, n = 3 for Docetaxel, Docetaxel + ABT263 and Docetaxel + S63845 from one experiment). The p-values were determined by two Way Anova followed by Tukey's multiple comparison test. The whole Anova results were given in the Supplementary Data 4. Data are represented as mean ± SD. **b,** Zoom out of pictures related to immune subsets infiltrating the prostate tumors in Supplementary Figure8f (Scale bar 300µm). Dashed squares represent areas visualized in Supplementary Figure8f. Data are representative of one experiment. Source data are provided as a Source Data file.

Supplementary Table 1 : List of available database used for out-of-clustering analysis

Reference	*#Samples*	*Description*	*PMID*
GSE115301	480	10 sc-RNAseq of ER:RasG12V IMR90 (+4OHT) co-culture with GFP+ cells	31018144
	141	smart-Seq2 of 100 growing and 41 senescent IMR90	
GSE61130	6	Non-senescent cells (EV - 4OHT) and Senescent cells (EV + 4OHT) IMR90 ER:RAS cells	26280535
GSE130727	37	WI-38, IMR-90, HUVEC, HAEC cells characterized by replicative exhaustion, ionizing radiation or doxorubicin treatment and expression of HRASG12V.	31251810
GSE102639	10	A549 cells treated with alisertib, barasertib, tozasertib or etoposide and untreated	29045843
GSE132369	6	Proliferating or senescent (IR) profiling of IIMR90 cells	32001510
E-MTAB-9970	60	13 cancer cell lines treated with a lisertib or etopside or proliferative	34320349
GSE98440	8	proliferative and senescent MSC cell line	29706538
	6	proliferative and senescent HUVEC cell line	
	8	proliferative and senescent IMR90 cell line	
GSE109270	12	PDX2 KRAS mut NSCLC tumors treated with vehicle or with SHP099	29808006
GSE158743	10	U2OS cells 7 days post IR or untreated	33459422

Supplementary Table 2: list of primer sequences used for RT-qPCR.

All the primers were purchased from Sigma Aldrich

Primer	Sequence	Purpose
H_MCL1 Fw	CCA AGA AAG CTG CAT CGA ACC AT	RT-qPCR
H_MCL1 Rev	CAG CAC ATT CCT GAT GCC ACC T	RT-qPCR
H_BCL2 Fw	GATGTGATGCCTCTGCGAAG	RT-qPCR
H_BCL2 Rev	CATGCTGATGTCTCTGGAATCT	RT-qPCR
H_E Cadherin Fw	CGAGAGCTACACGTTCACGG	RT-qPCR
H_E Cadherin Rev	GGGTGTCGAGGGAAAAATAGG	RT-qPCR
H_N Cadherin Fw	TCAGGCGTCTGTAGAGGCTT	RT-qPCR
H_N Cadherin Rev	ATGCACATCCTTCGATAAGACTG	RT-qPCR
H_FN1 Fw	CGGTGGCTGTCAGTCAAAG	RT-qPCR
H_FN1 Rev	AAACCTCGGCTTCCTCCATAA	RT-qPCR
H_Snai1 Fw	TCGGAAGCCTAACTACAGCGA	RT-qPCR
H_Snai1 Rev	AGATGAGCATTGGCAGCGAG	RT-qPCR
H_Snai2 Fw	CGAACTGGACACACATACAGTG	RT-qPCR
H_Snai2 Rev	CTGAGGATCTCTGGTTGTGGT	RT-qPCR
H_Twist Fw	GCAAGAAGTCGAGCGAAGAT	RT-qPCR
H_Twist Rev	GCTCTGCAGCTCCTCGAA	RT-qPCR
H_p16 Fw	ACCAGAGGCAGTAACCATGC	RT-qPCR
H_p16 Rev	TGTCGTTCGCGGGCGCAACTG	RT-qPCR
H_p21 Fw	TGTCCGTCAGAACCCATGC	RT-qPCR
H_p21 Rev	AAAGTCGAAGTTCCATCGCTC	RT-qPCR
H_p27 Fw	TAATTGGGGCTCCGGCTAACT	RT-qPCR
H_p27 Rev	TGCAGGTCGCTTCCTTATTCC	RT-qPCR
H_PAI-1 Fw	TGCTGGTGAATGCCCTCTACT	RT-qPCR
H_PAI-1 Rev	CGGTCATTCCCAGGTTCTCTA	RT-qPCR
H-b Actin Fw	GAGAAAATCTGGCACCACACC	RT-qPCR
H_b Actin Rev	ATACCCCTCGTAGATGGGCAC	RT-qPCR
m_MCL1 Fw	AAGAGGCTGGGATGGGTTTGT	RT-qPCR
m_MCL1 Rev	AGTCCCCTATTGCACTCACAAG	RT-qPCR
m_BCL2 Fw	TACCGTCGTGACTTCGCAGAG	RT-qPCR
m_BCL2 Rev	GGCAGGCTGAGCAGGGTCTT	RT-qPCR
R18s Fw	ACCGCAGCTAGGAATAATG	RT-qPCR
r18s Rev	GCCTCAGTTCCGAAAACCA	RT-qPCR
GADPH Fw	AGGTCGGTGTGAACGGATTTG	RT-qPCR
GADPH Rev	TGTAGACCATGTAGTTGAGGT	RT-qPCR
m_PAI-1 Fw	GCCAGATTTATCATCAATGA	RT-qPCR
m_PAI-1 Rev	GGAGAGGTGCACATCTTTCTCAAAG	RT-qPCR
m_p27-1 Fw	AACGTGCCGAGTGTGTCTAACGG	RT-qPCR
m_p27-1 Rev	CCTCTAGGGGTTTGTGATTCT	RT-qPCR
m_Perforin-1 Fw	AGCACAAGTTCGTGCCAGG	RT-qPCR
m_Perforin-1 Rev	GCGTCTCTCATTAGGGAGTTTTT	RT-qPCR
