# Supplementary material for: Author Correction: Single-cell transcriptomics identifies Mcl-1 as a target for senolytic therapy in cancer
Source: Nat Commun. 2023 Jul 20;14:4386. doi: 10.1038/s41467-023-40080-9 (PMC10359282; doi:10.1038/s41467-023-40080-9)

# Figure 4c 3 biological replicates

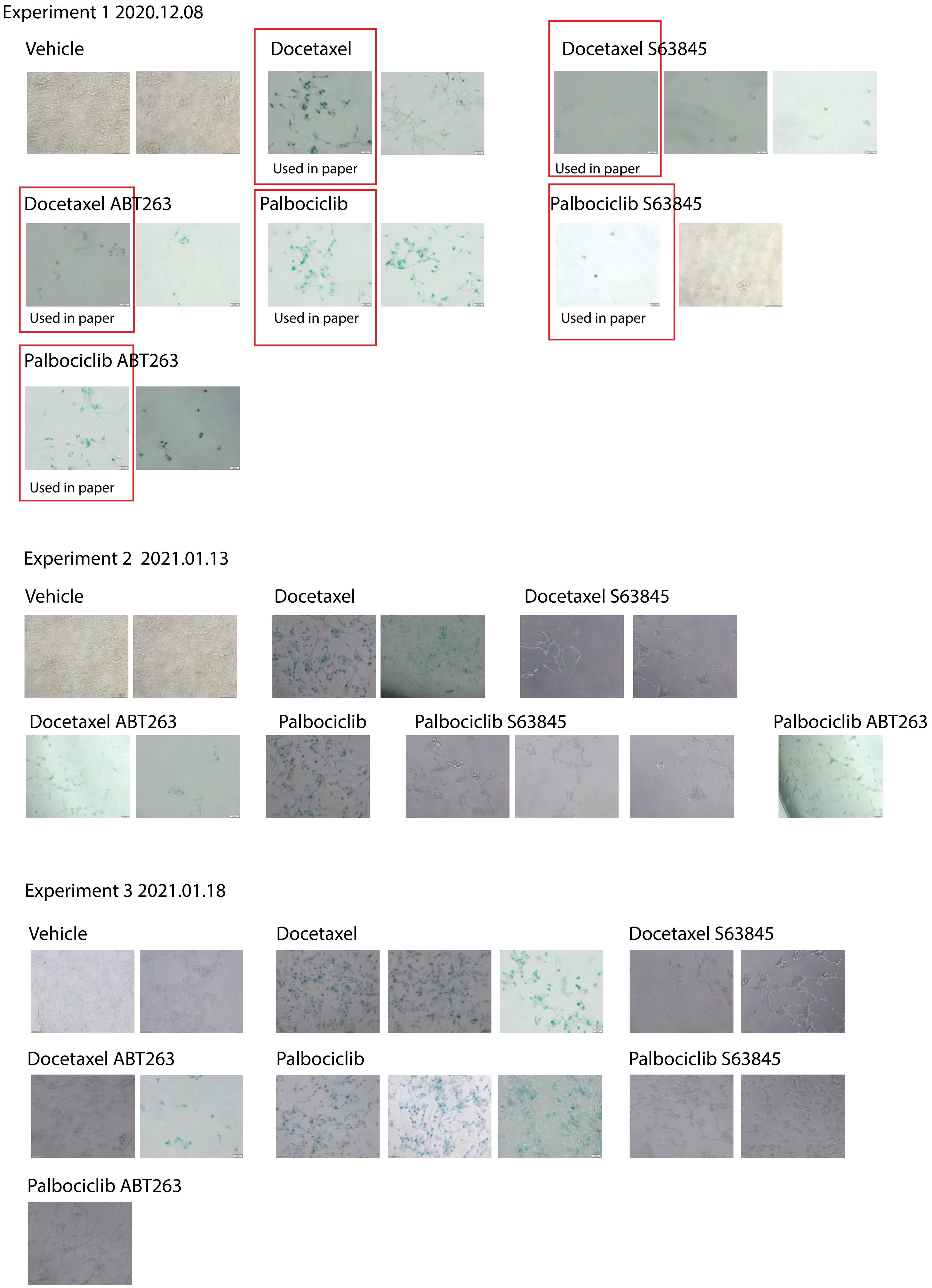

# Supplementary Figure 8c Mice sacrified at 2021.10.25

Biological replicates done with different mice. Staining performed on the same day

ID number of each mice is showed as label of each image

## p16

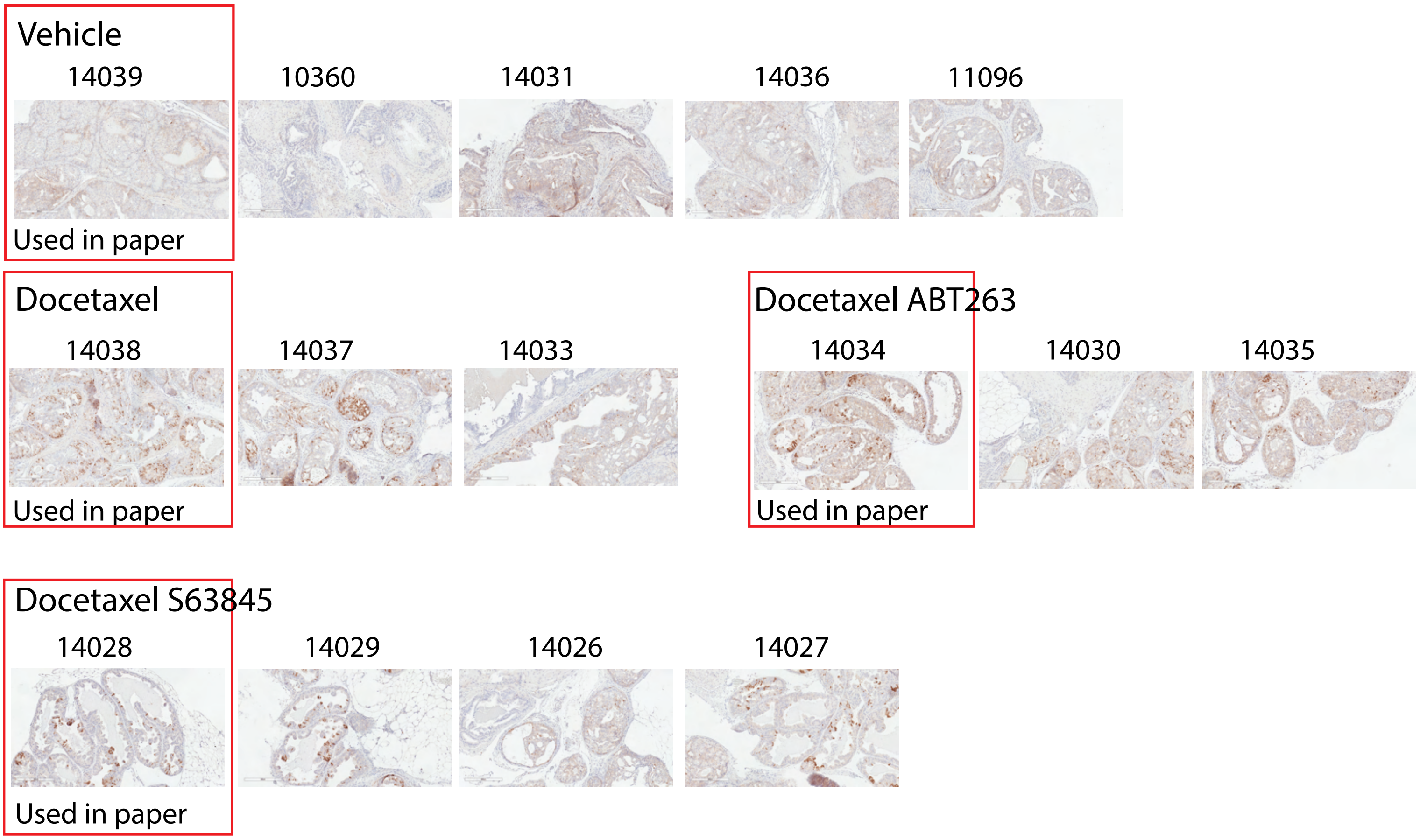

# Supplementary Figure 8

Mice sacrified at 2021.10.25 Biological replicates done with different mice. Staining performed on the same day
ID number of each mice is showed as label of each image

## Cleaved Caspase 3

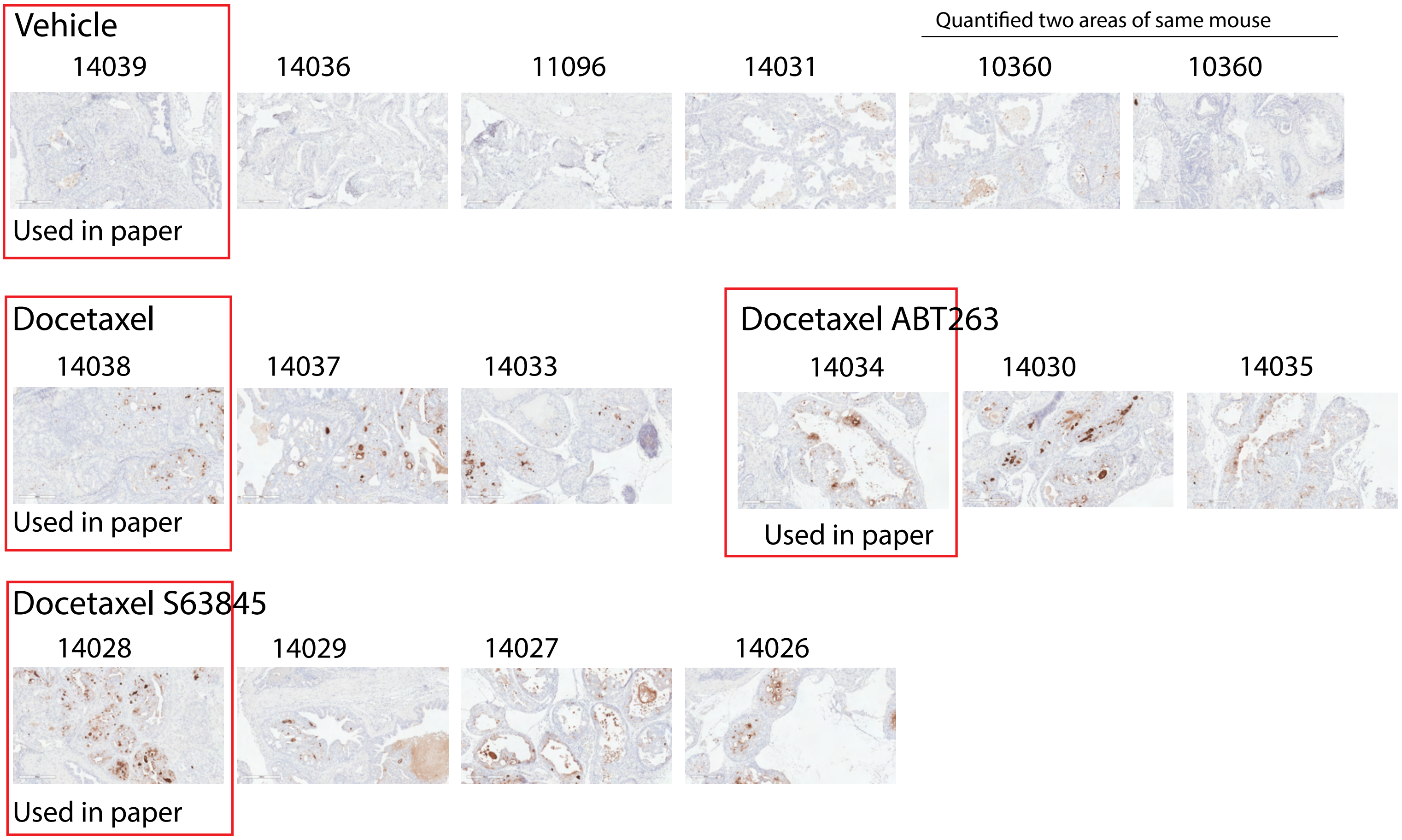


## KI67

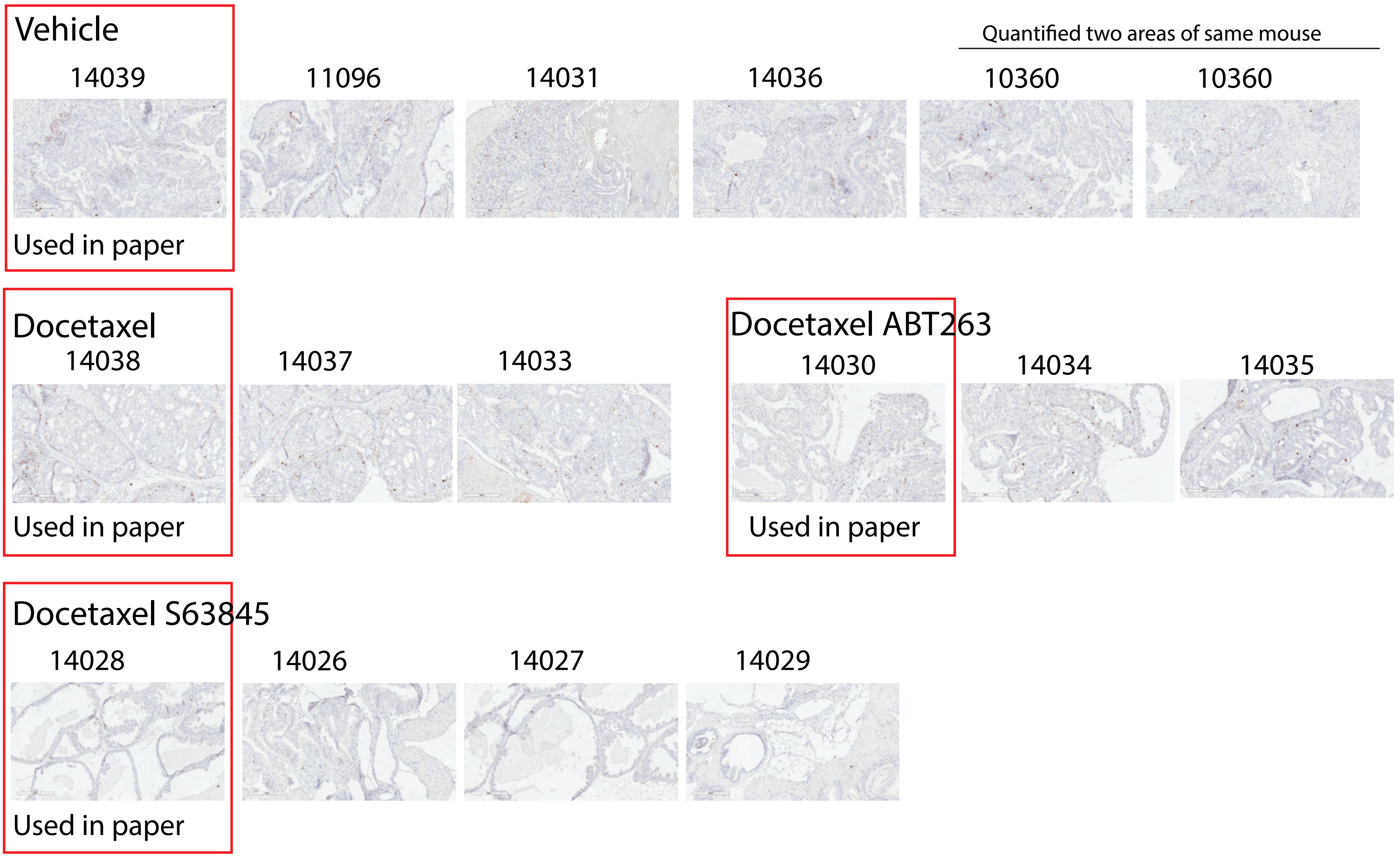

Supplementary Figure 8f

Mice sacrified at 2021.10.25 Biological replicates done with different mice. Staining performed on the same day
ID number of each mice is showed as label of each image

F4/80 (macrophages)

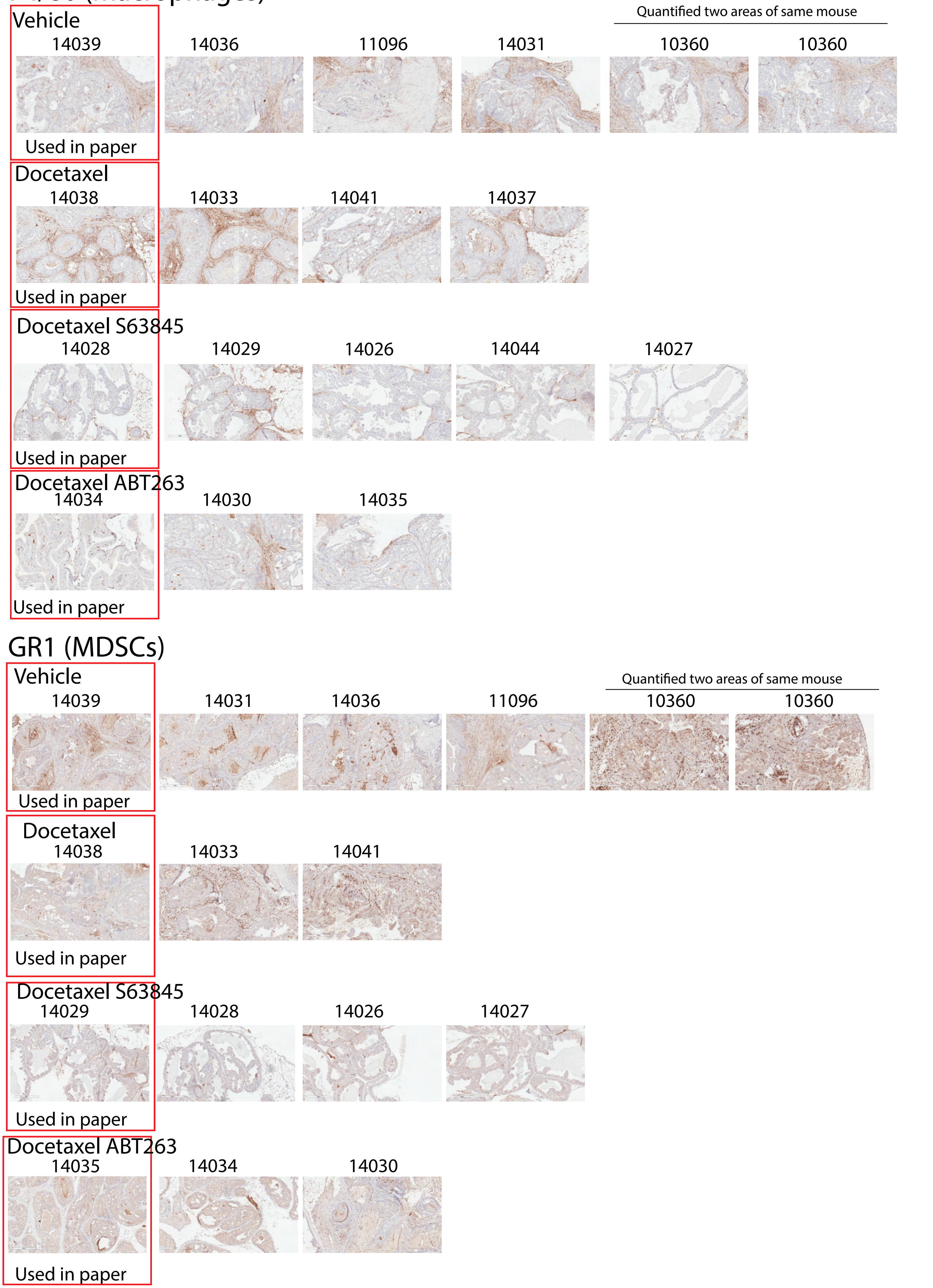

Supplement: Supplementary file 2 — Merged Replicate Data [file 41467_2023_40080_MOESM2_ESM.pdf]
